# Adaptive cold-environmental strategies of *Lonicera caerulea*: synergistic regulation of anthocyanin and iridoid pathways in cold tolerance and fruit ripening

**DOI:** 10.1093/hr/uhag198

**Published:** 2026-05-20

**Authors:** Tianyi Wang, Qing Yang, Hongyan Cao, Tingting Du, Baoan Wang, Yuxin Fan, Cai Qin, Yadong Duan, Yujie Fu, Dong Meng

**Affiliations:** College of Forestry, Beijing Forestry University, Beijing 100000, China; Beijing Key Laboratory of Efficient Biosynthesis of Plant Secondary Metabolites, Beijing Forestry University, Beijing 100083, China; College of Forestry, Beijing Forestry University, Beijing 100000, China; Beijing Key Laboratory of Efficient Biosynthesis of Plant Secondary Metabolites, Beijing Forestry University, Beijing 100083, China; College of Forestry, Beijing Forestry University, Beijing 100000, China; Beijing Key Laboratory of Efficient Biosynthesis of Plant Secondary Metabolites, Beijing Forestry University, Beijing 100083, China; College of Forestry, Beijing Forestry University, Beijing 100000, China; Beijing Key Laboratory of Efficient Biosynthesis of Plant Secondary Metabolites, Beijing Forestry University, Beijing 100083, China; College of Horticulture, China Agricultural University, Beijing 100193, China; College of Forestry, Beijing Forestry University, Beijing 100000, China; Beijing Key Laboratory of Efficient Biosynthesis of Plant Secondary Metabolites, Beijing Forestry University, Beijing 100083, China; College of Forestry, Beijing Forestry University, Beijing 100000, China; Northeast Institute of Geography and Agroecology, Chinese Academy of Sciences, Harbin 150081, China; College of Forestry, Beijing Forestry University, Beijing 100000, China; Beijing Key Laboratory of Efficient Biosynthesis of Plant Secondary Metabolites, Beijing Forestry University, Beijing 100083, China; College of Forestry, Beijing Forestry University, Beijing 100000, China; Beijing Key Laboratory of Efficient Biosynthesis of Plant Secondary Metabolites, Beijing Forestry University, Beijing 100083, China

## Abstract

Understanding how plants adapt to extreme cold and ripen fruit is a key question in plant biology. *Lonicera caerulea* (blue honeysuckle), a cold-tolerant berry species native to the northern temperate zone, is valued as a ‘noble fruit’ for its rich nutrient profile and serves as an ideal model for studies due to its unique ability to thrive in low temperatures and ripen fruit during early spring. Here, we generated a chromosome-level genome assembly for wild *L. caerulea* from the Changbai Mountain region. We integrated it with transcriptomic and metabolomic analyses to explore its molecular cold adaptation mechanisms. We found that *L. caerulea* achieves cold tolerance and fruit ripening through dynamic regulation of two important metabolic pathways. Under cold stress, the LcODO1/LcMYB15–LcSLAS module promotes iridoid accumulation, while the LcbHLH62/LcMYB3R5–LcF3GT module enhances anthocyanin biosynthesis, jointly increasing plant cold resistance. During fruit maturation, metabolite profiles show dynamic reprogramming: anthocyanin levels rise in both pulp and peel, while the content of bitter iridoids markedly decreases in the pulp. This work provides a comprehensive genomic framework for understanding cold adaptation mechanisms, offering valuable gene resources and a theoretical foundation for improving cold resistance and fruit quality in breeding programs.

## Introduction

Plant growth and development are continually challenged by complex environments and various biotic and abiotic stresses. These include biological stresses such as fungi pathogens and insect herbivores, as well as abiotic stresses like extreme temperatures and drought. To cope with these pressures, plants have evolved a wide array of adaptive strategies, ranging from morphology and structure changes to the synthesis and accumulation of specialized bioactive metabolites. For instance, plants in arid regions with limited rainfall often develop succulent organs, which help them survive under intense sunlight and drought conditions [1]. Heterophyllous plants cope with waterlogging by regulating the development of aquatic vs terrestrial leaf types [2]. Cold-adapted species such as winter rye (*Secale cereale*), Mongolian sand acanthopanax (*Ammopiptanthus mongolicus*), and Tangut rhodiola (*Rhodiola algida* var. tangutica) produce antifreeze proteins to withstand subzero-temperature environments [3–5]. Among chemical defense strategies, the synthesis and accumulation of diverse secondary metabolites play a central role in enhancing plant stress resistance. For example, phenolic compounds strengthen the cell wall structure and improve disease resistance [6]; terpenoids serve as signaling molecules to induce systemic defense response [7, 8]; and alkaloids can disrupt pests’ nervous system, reducing herbivory [9, 10].

*L. caerulea*, commonly known as blue honeysuckle, is a perennial shrub belonging to the Caprifoliaceae family. It is widely distributed across high-latitude regions of the Northern Hemisphere and has a long history of medicine and edible use. Historically, its fruits were fermented into alcoholic beverages, and its young branches and leaves were also used in traditional herbal remedies [11]. The fruits develop a distinctive double-berry structure from paired flowers, with unique pigmentation and flavor (Fig. 1A). They are nutrient-rich and considered the ‘third-generation berries’ due to their notable health benefits such as reducing blood pressure and lipid levels [12]. Anthocyanins contribute to fruits’ strong deep coloration and potent antioxidant properties [13], while iridoids—bitter-tasting secondary metabolites—are key determinants of flavor and medicinal value [14]. These compounds also exhibit important pharmacological activities, such as anti-inflammatory and antiviral effects, commonly associated with *Lonicera* species [15, 16].

**Figure 1:**
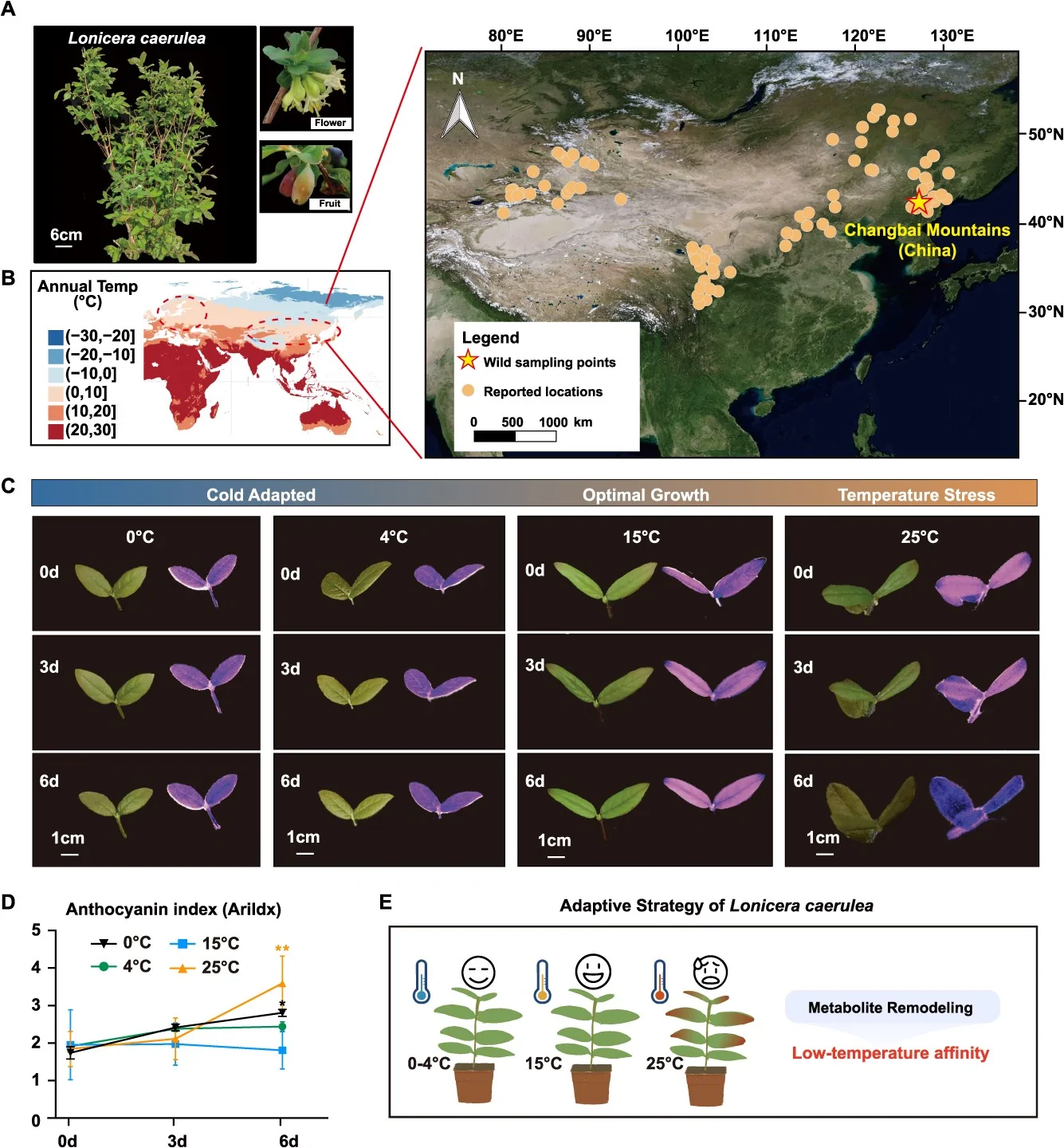
Effects of temperature on the phenotype and metabolite accumulation in *L. caerulea*. (A) Morphological characteristics of *L. caerulea*. Bar = 6 cm. (B) Global distribution of wild *L. caerulea* populations overlaid with annual average temperature data. (C) Leaf phenotypes under different temperature gradients. Bar = 1 cm. (D) Anthocyanin index (Arildx) of leaves under different temperature gradients. (E) Schematic representation of the low-temperature affinity of *L. caerulea*.

What makes *L. caerulea* especially intriguing is its exceptional cold tolerance: it can survive temperatures as low as −50°C without damage and fruit successfully in harsh cold climates across Europe, Asia, and North America. In China, it is mainly found in forests at high-altitude areas between 40°Nand 53°N, with the richest natural populations located in the Greater and Lesser Xing’an Mountains and the Changbai Mountain region of Northeast China [14]. The Changbai Mountains serve as an ecological barrier for Northeast Asia and are recognized as a ‘genetic treasure trove’ among regions at similar latitudes globally. Wild *L. caerulea* populations in this area have not undergone artificial domestication, preserving ancient genetic lineages and diverse traits shaped by long-term natural selection under extreme environmental pressures such as cold temperatures and high radiation [12]. These wild populations thus provide a unique window into the evolutionary processes underlying cold tolerance, including both the heritable traits that enable survival across generations in persistently cold environments and the rapid physiological mechanisms that are activated when temperatures drop. Understanding cold tolerance in plants requires consideration of processes operating at timescales. Over evolutionary time, populations exposed to chronic cold have accumulated heritable genetic changes, such as gene family expansions and constitutive metabolic adjustments, that enhance survival and reproduction. This capacity is what we refer to as cold adaptation. Even cold-adapted species also cope with short-term temperature fluctuations. The molecular and metabolic responses triggered by such temperature drops constitute what we term cold stress response, including rapid transcriptional reprogramming and transient metabolite accumulation. Critically, the capacity to mount an effective short-term stress response is itself an adaptive trait, and the acutely activated pathways are likely key components of the adaptive strategy repeatedly deployed in nature. Despite the roles of anthocyanins in fruit pigmentation and iridoids in medicinal applications, how these two metabolite classes function together during temperature challenges, and whether their coordinated regulation both serves protective functions and contributes to long-term cold adaptation, remain poorly understood in *L. caerulea* [17, 18]. To address this knowledge gap, the present study aims to systematically elucidate the molecular mechanisms underlying cold adaptation in *L. caerulea*, with a focus on the biosynthesis and regulatory roles of key bioactive metabolites. Using PacBio HiFi and Hi-C technologies, we assembled a high-quality, chromosome-level genome of wild *L. caerulea* collected from the Changbai Mountain region. By integrating multi-omics approaches, including genomics, transcriptomics, and metabolomics, we investigated how the capacity for rapid metabolic reprogramming may contribute to the species’ long-term persistence in cold environments. Our findings reveal a coordinated regulatory mechanism of anthocyanins and iridoids that contributes to environmental adaptation in *L. caerulea*. This research not only advances the theoretical understanding of the plant’s adaptive strategies but also offers valuable genomics resources for future breeding aimed at improving stress resistance and enhancing the nutritional and medicinal value of this species.

## Results

### Impact of cold environment on the distribution and metabolite accumulation in *L. caerulea*

*L. caerulea* demonstrates remarkable adaptability to cold environments. Its fruits can mature at relatively low temperature (LT) conditions (usually in May), ripening earlier than those of apples, raspberries, blueberries, kiwis, and other common fruits (Fig. S1). Comparative studies show that, under identical temperature conditions, *L. caerulea* exhibits significantly greater tolerance to cold temperatures (Fig. S2). To determine the optimal temperature range for *L. caerulea*, data from global distribution areas were combined with average annual temperature datasets (Fig. 1B). Results showed that it is distributed in regions with an average temperature ranging from −10 to 10°C. In China, it is predominantly found in northern Xinjiang, the eastern edge of the Qinghai-Tibet Plateau, northern North China, and the Changbai Mountains in the Northeast (Fig. 1B). These findings highlighted temperature as a key factor influencing the geographic distribution and growth patterns of *L. caerulea*.

To further investigate cold tolerance thresholds, *L. caerulea* was grown at four representative temperatures: 0°C (freezing point), 4°C (standard condition), 15°C (critical temperature), and 25°C (regular temperature) [19]. Morphological analysis (Fig. 1C and D) showed that leaves grown at 25°C developed a reddish-brown discoloration from the tip to the petiole, with a significant increase in the anthocyanin index by the sixth day, indicating rapid anthocyanin accumulation. At 15°C, plants grew normally with minimal anthocyanin index fluctuation. At 4°C, leaves showed no obvious color change, though a modest rise in the anthocyanin index was observed on the third and sixth days. Remarkably, at 0°C, plants exhibited strong physiological stability. Based on visual observations, there were no visible chilling injury symptoms (including color change or wilting), and corresponding electrolyte leakage measurements remained stable. Despite this physiological robustness, a significant anthocyanin accumulation, as indicated by an increase in anthocyanin index, was detected by day 6. These results suggest that *L. caerulea* actively accumulates anthocyanins under cold stress, likely as a protective metabolic response. The observed range of phenotypic responses and metabolic adjustments across these temperatures highlights *L. caerulea*’s distinct low-temperature affinity and its physiological plasticity to tolerate environmental stress. This physiological plasticity in cold environments likely drives the evolution of its specialized adaptive strategies (Fig. 1E).

### High-quality assembly and annotation of the wild *L. caerulea* genome

Wild populations of *L. caerulea* preserve a full spectrum of genetic diversity shaped by natural evolution, including traits critical for environmental adaptation. To obtain genetic foundations, a high-quality analysis genomic data for Changbai Mountain wild *L. caerulea* was performed. The genome was sequenced and assembled using Illumina, PacBio HiFi, and Hi-C technologies. Based on k-mer depth distribution analysis (*K* = 17, depth = 63) (Fig. S3), the genome size was estimated to be about 829.48 Mb, with a heterozygosity of 1.26% and a repeat content of 63.22% (Table S1). During this process, we generated 75.89 Gb of Illumina reads, 44.99 Gb of PacBio HiFi reads, and 150.73 Gb of Hi-C data, achieving ~55× coverage of the *L. caerulea* genome (Table S2).

The final assembly was anchored to nine chromosomes (Fig. 2A), with a total size of 806.04 Mb, a contig N50 of 69.46 Mb, and a GC content of 37.67% (Fig. 2B). Assembly quality was validated using Benchmarking Universal Single-Copy Orthologs (BUSCO) and the Core Eukaryotic Genes Mapping Approach (CEGMA). BUSCO analysis identified 1614 complete single-copy orthologs, with 99.4% completeness (Table S3). CEGMA analysis confirmed a completeness of 96.77% based on 242 out of 248 core eukaryotic genes that were successfully assembled (Table S4). The alignment rate of small fragment reads yielded ~98.98%, with 99.95% coverage (Table S5). Additionally, SNP calling revealed 4 317 351 SNPs, of which 4 316 811 were heterozygous (0.00583%) and 540 were homologous (0.000001%) (Table S6). Repetitive sequences constituted 55.18% of the genome, with long terminal repeats (LTRs) representing 41.20% (Table S7).

**Figure 2:**
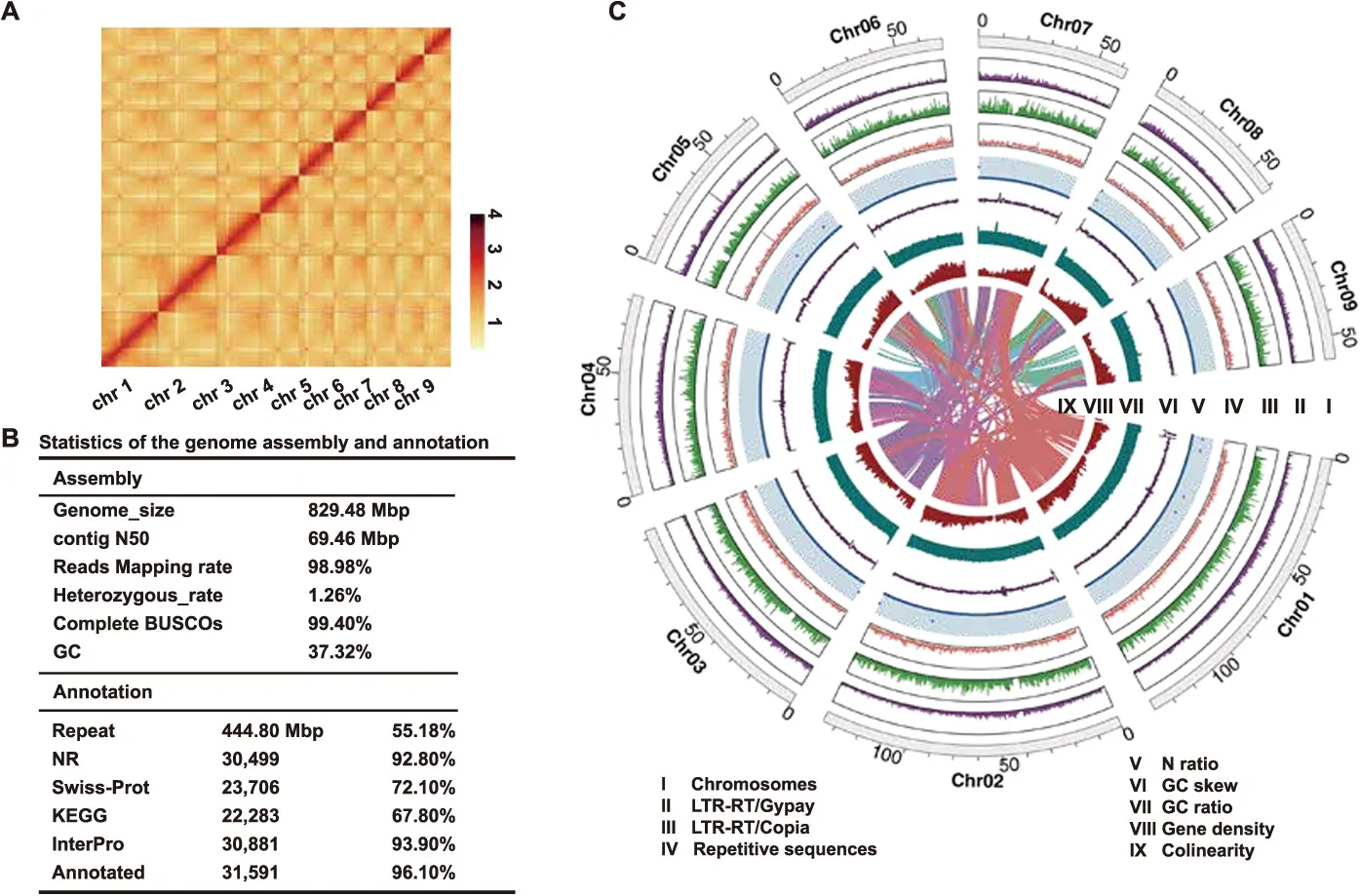
Overview of the genome assembly and annotation of wild *L. caerulea*. (A) Hi-C map of wild *L. caerulea* genome showing a high-quality assembled genome. (B) Summary statistics of the genome assembly and functional annotation. (C) Overview of the assembled *L. caerulea* genome structure and features.

To support gene annotation, we collected transcriptomic data from five tissue types: root, stem, leaf, young fruit, and mature fruit. Combined with gene structure *de novo* prediction and the identification of homologous species, we annotated 32 873 protein-coding genes (Table S8). Of these, 96.10% were predicted functional annotations, with 92.80% annotated in the NR database, 72.10% in Swiss-Prot, 67.80% in KEGG, and 93.90% in InterPro (Fig. 2B). Additionally, non-coding RNAs were annotated, including 2417 miRNAs and 1024 tRNAs (Table S9). Collectively, these results demonstrated the successful assembly of a high-quality chromosome-level genome for wild *L. caerulea* (Fig. 2C). The high completeness accuracy of this genomic resource lays a robust foundation for investigating the species’ evolutionary history and adaptive responses to extreme environments.

### Comparative genomics analysis reveals the genomic basis of *L. caerulea*’s adaptation to the environment

To elucidate the genomic basis of cold adaptation in *L. caerulea*, we performed comparative genomics analyses using a diverse panel of species with varying phylogenetic relationships and temperature adaptations. We chose two cold-adaptated species within the same family, *Lonicera japonica* and *Triplostegia glandulifera*, selected as close relatives with similar ecological niches. Additionally, we included seven species occupying different climatic zones: *Nymphoides indica* and *Centella asiatica* (warm, humid tropical to subtropical climates); *Scaevola taccada* (a typical tropical coastal species); *Panax vietnamensis* (mild habitats at low latitudes and high altitudes); and *Panax stipuleanatus*, *Sanicula chinensis*, and *Angelica sinensis* (temperate to subtropical climates with limited cold tolerance).

The *L. caerulea* genome encoded 32 873 genes, among which 1145 genes belong to species-specific orthogroups via comparison with other species. Phylogenetic analysis based on 1104 shared single-copy gene families (Fig. 3A) revealed that Caprifoliaceae was closely related to Menyanthaceae and Goodeniaceae, with divergence from other plant families estimated at ~67.98 million years ago. Notably, *L. caerulea* and *L. japonica* diverged around 11.43 million years ago during the Miocene (the first period of the Neogene). Fourfold synonymous third-codon transversion (4DTv) analysis showed two peaks that almost perfectly overlap in both *L. caerulea* and *L. japonica*, indicating two whole-genome duplication (WGD) events in their shared ancestry (Fig. 3B). Based on the peaks, divergence time estimates, and comparison with previously reported polyploidy events in angiosperms, the older peak corresponds to the γ WGD event, while the more recent peak represents a Caprifoliaceae-specific WGD event, which has been reported in related studies of other species in the Caprifoliaceae family [20–22]. Additionally, syntenic analyses confirmed numerous conserved genomic blocks among *L. caerulea*, *L. japonica*, and *Vitis vinifera* (Fig. 3C). The syntenic depth ratio between *L. caerulea* and *V. vinifera* was 2:1, as well as between *L. japonica* and *V. vinifera*, while *L. caerulea* and *L. japonica* exhibited a 1:1 ratio (Fig. 3D). These findings provided strong evidence for WGD events. Gene family analysis revealed that the five closely related species had different numbers of families. *N. indica, S. taccada,* and the Caprifoliaceae family had higher numbers of 19 452, 14 407, and 19 452 gene families, respectively, while *L. caerulea*, *L. japonica*, and *T. glandulifera,* 790, 1161, and 1268 gene families, respectively (Fig. 3E). These lineage-specific gene families are likely associated with the evolution of unique traits and environmental adaptations.

**Figure 3:**
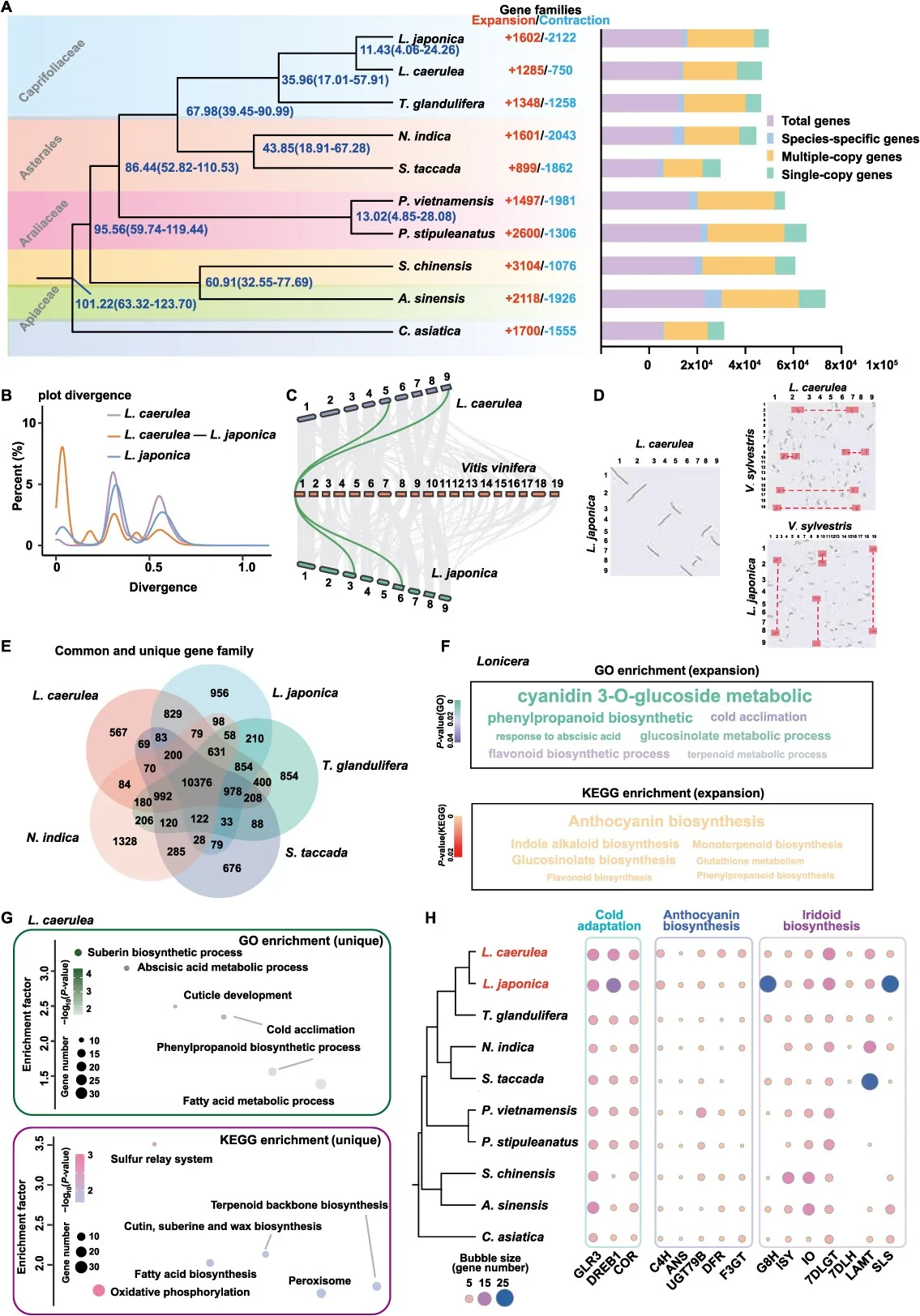
Phylogenetic and comparative genomic characteristics of *L. caerulea*. (A) Phylogenetic tree of *L. caerulea* and nine other plant species. Expanded gene families are indicated by positive values (+), and contracted gene families by negative values (-). Estimated divergence times (in millions of years ago) are shown at each node, with 95% confidence intervals in parentheses. The bar chart represents the comparative analysis of homologous gene families. (B) 4DTv analysis. (C and D) Synteny blocks among chromosomes of *L. caerulea*, *L. japonica*, and *V. sylvestris*. (E) Venn diagram showing the shared and unique gene families between *L. caerulea* and four closely related species (*L. japonica*, *T. glandulifera*, *S. taccada*, and *N. indica*). (F) Enriched pathways of expanded gene families in the genus *Lonicera*. (G) Enriched pathways of species-specific gene families in *L. caerulea*. (H) Heatmap showing the expansion of cold adaptation genes, anthocyanin biosynthesis genes, and iridoid biosynthesis genes in the genus *Lonicera* compared to other species.

The expansion and contraction of gene families are known to contribute to gene dosage effects that influence adaptive phenotypes such as stress resistance and species development, providing a molecular basis for their ability to adapt to complex environments [23, 24]. In Caprifoliaceae, the expanded gene families were significantly enriched in pathways related to secondary metabolite biosynthesis (e.g. indole alkaloid, cyanamide acid, and phenylpropanoid biosynthesis) as well as in biological processes related to abiotic stress response, including ABA signaling, and salt stress (Fig. S4A). The expansion of these gene families forms the basis for the rich array of active metabolites found in Caprifoliaceae. In the *Lonicera* genus, gene family expansions were particularly enriched in gene ontology (GO) processes such as cold acclimation, flavonoid biosynthesis, terpenoid metabolism, and cyanidin 3-O-glucoside metabolic pathways (Fig. 3F). This supported increased secondary metabolite capacity and cold responsiveness. Notably, compared to other *Lonicera* species, *L. caerulea* exhibited gene families involved in isoflavonoid biosynthesis, RNA degradation, ribosome function, and exosome activity (Fig. S4B). Additional significant enrichment was observed in pathways such as oxidative phosphorylation, terpenoid backbone biosynthesis, fatty acid metabolism, and phenylpropanoid biosynthesis (Fig. 3G). These genomic features were likely integral to the plant’s robust metabolic activity and cold adaptation. Cold tolerance in plants is often mediated by the DREB-COR signaling pathway and the accumulation of protective secondary metabolites [25]. Consistent with this, GLR3, DREB1, and COR gene families showed higher copy numbers in *Lonicera* compared with several related species (Fig. 3H; Table S11). For example, the *L. caerulea* genome contains 11 GLR3 genes, 11 DREB1 genes, and 8 COR genes. Notably, the *L. caerulea* genome showed a significant enrichment in anthocyanin biosynthesis pathways—important for fruit pigmentation—as well as those linked to terpenoid and indole alkaloid production, potentially connected to the synthesis of pharmacologically relevant iridoids. Within the *L. caerulea* genome, enzyme genes involved in anthocyanin biosynthesis included 5 C4H, 1 ANS, 4 UGT79B, 5 F3GT, and 5 DFR. Additionally, enzymes related to iridoid biosynthesis included 4 G8H, 4 ISY, 5 IO, 12 7DLGT, 2 7DLH, 8 LAMT, and 6 SLS. These genomic features likely facilitate the biosynthetic efficiency and diversity of anthocyanins and iridoids, which are likely key contributors to *L. caerulea*’s cold tolerance and medicinal properties.

### Temporal dynamics of bioactive components in response to cold stress

Preliminary experiments showed that cultivation at 4 and 0°C did not result in obvious morphological changes in *L. caerulea* leaves. To explore the plant’s metabolic response under more extreme conditions, a −4°C cold stress treatment was applied. As exposure time increased, the plants exhibited progressively severer freezing damage: after 12 h, wilting began in upper leaves; by 24 h, it spread to middle leaves; and after 48 h, widespread leaf wilting was observed at most leaves, accompanied by a significant increase in electrical conductivity (Fig. 4A). Metabolomic analysis identified 3374 metabolites (Fig. 4B). Over time, a clear shift in metabolite profiles was observed: the relative abundance of primary metabolites showed a decreasing trend (56.68% at 0, 57.40% at 12, 48.26% at 24, and 51.79% at 48 h), while secondary metabolites increased (40.25% at 0, 41.48% at 12, 48.19% at 24, and 44.08% at 48 h). This shift indicated that the reallocation of metabolic flux was toward secondary metabolism under stress conditions (Fig. 4C). Secondary metabolites were classified into three major categories: phenylpropanoids, isoprenoids, and alkaloids. Among these, terpenoids (a subgroup of isoprenoids) and flavonoids (a subgroup of phenylpropanoids) dominated the profile, underscoring their potential roles in cold adaptation (Fig. 4D).

**Figure 4:**
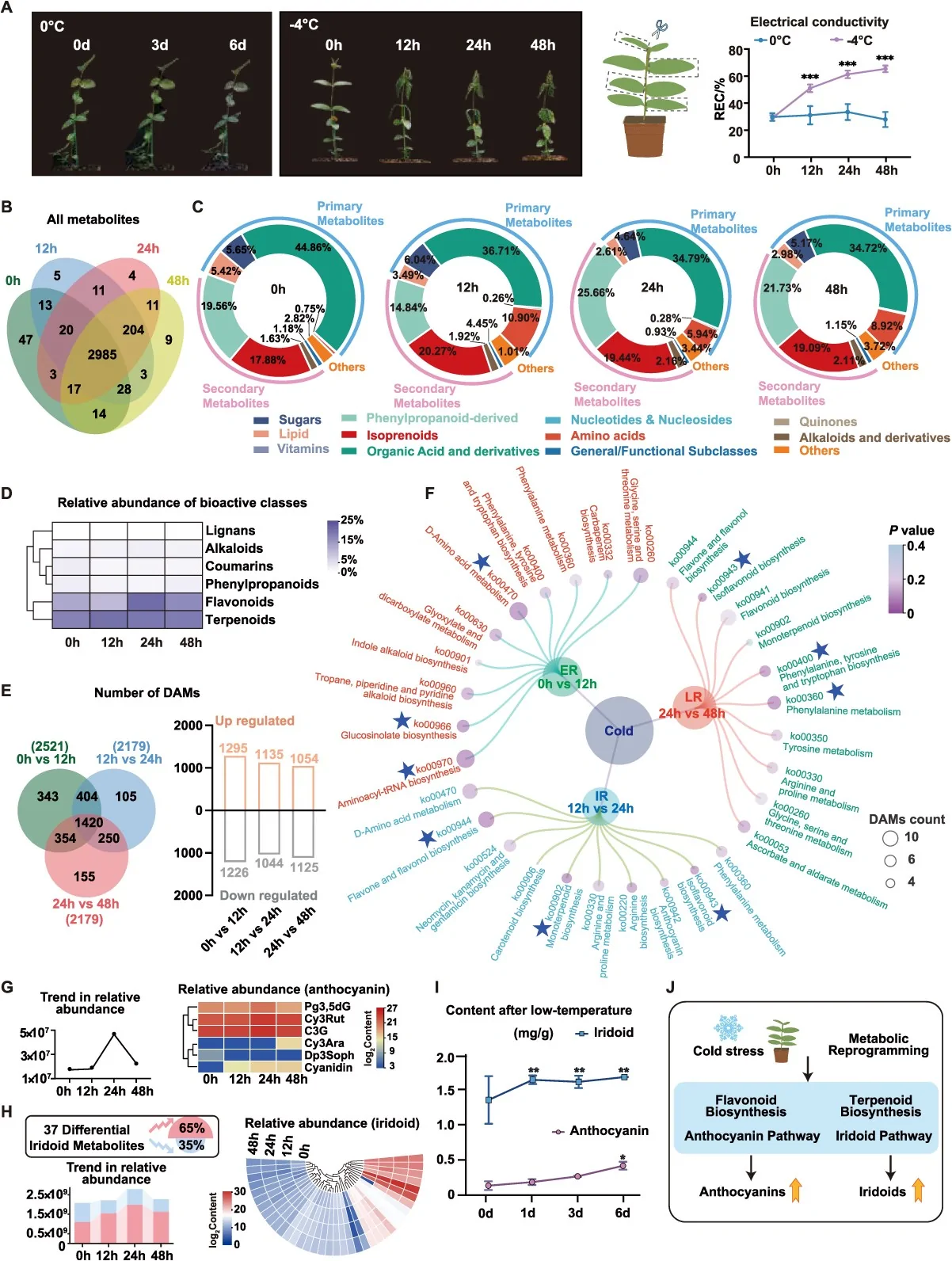
Metabolic reprogramming of *L. caerulea* in response to cold stress. (A) Phenotypic changes of *L. caerulea* under 0°C (0d, 3d, 6d) and −4°C (0, 12, 24, 48 h) treatments, and the REC of leaves at different time points is shown to reflect membrane damage. (B) Venn diagram showing the distribution of all detected metabolites at four time points, 0, 12, 24, and 48 h, under −4°C treatment. (C) Proportional distribution of primary metabolites, secondary metabolites, and other metabolites at 0, 12, 24, and 48 h. (D) Heatmap showing the relative abundance of different bioactive metabolite classes at 0, 12, 24, and 48 h. (E) Statistical analysis of the number of DAMs at different comparison time points (0 vs 12 h, 12 vs 24 h, 24 vs 48 h), including upregulated and downregulated DAMs. (F) KEGG enrichment analysis of DAMs at different comparison time points. The size of the circles represents the number of DAMs, and the color gradient corresponds to statistical significance (*P-*value). (G) Trend analysis of the relative abundance of anthocyanin metabolites and heatmap of their specific composition. (H) Statistical analysis of the number and relative abundance trend of iridoid metabolites, and the proportional distribution of different iridoid metabolites. (I) Accumulation dynamics of total anthocyanin and iridoid content in *L. caerulea* leaves after low-temperature treatment. (J) Proposed schematic model illustrating the metabolic reprogramming mechanism of *L. caerulea* in response to cold stress, involving the flavonoid biosynthesis (anthocyanin pathway) and terpenoid biosynthesis (iridoid pathway).

Dynamic metabolite changes were further analyzed using differential accumulation metabolite (DAM) profiling (Fig. 4E). Compared with 0 h, 2521 DAMs were identified at 12 h (1295 upregulated and 1226 downregulated). Between 12 and 24 h, 2179 DAMs were detected (1135 upregulated and 1044 downregulated), and from 24 to 48 h, another 2179 DAMs were found (1054 upregulated and 1125 downregulated). Pathway enrichment analysis showed that early-stage DAMs (0 vs 12 h) were significantly enriched in pathways such as D-amino acid metabolism, aminoacyl-tRNA biosynthesis, and glucosinolate biosynthesis—reflecting rapid activation of stress signaling. In the middle phase (12 vs 24 h), DAMs shifted toward flavone/flavonol biosynthesis, monoterpenoid biosynthesis, and isoflavonoid biosynthesis. During the late stage (24 vs 48 h), DAMs were predominantly enriched in phenylalanine metabolism, phenylalanine, tyrosine, and tryptophan biosynthesis, and continued isoflavonoid biosynthesis, suggesting the ongoing production of downstream defense metabolites (Fig. 4F). Across all stages, flavonoid and terpenoid biosynthesis pathways remained persistently active, highlighting their central role in *L. caerulea*’s cold stress adaptations. These findings closely align with previously identified genomic features of *L. caerulea*.

Building upon these broad metabolic shifts and pathway enrichments, our focused metabolome analysis further revealed the specific accumulation patterns of key secondary metabolites relevant to cold adaptation. Metabolome analysis further revealed six anthocyanin metabolites, with increasing abundance over time, especially pelargonidin-3,5-O-diglucoside chloride, cyanidin-3-O-rutinoside, and cyanidin-3-O-glucoside (Fig. 4G). Additionally, 37 iridoid metabolites were detected, with ~65% showing an upregulated trend. Loganic acid emerged as the most abundant iridoid (Fig. 4H). Quantification confirmed that both total anthocyanin and iridoid contents displayed significant increasing trends (Fig. 4I). Crucially, these specific metabolic accumulations were strongly corroborated by transcriptional analysis, which showed marked upregulation of genes in both anthocyanin and iridoid biosynthesis pathways under −4°C cold stress (Fig. 6). These results supported the conclusion that *L. caerulea* enhances the biosynthesis of these key secondary metabolites under cold stress (Fig. 4J), and such coordinated upregulation of both metabolites and their biosynthetic genes likely represent core components of the plant’s adaptive strategy to extreme temperatures.

### Genomic insights into metabolic dynamics during fruit ripening in *L. caerulea*

Plant adaptive strategies often involve coordinated adjustments in morphology, physiology, and reproductive processes. While cold tolerance mainly depends on organs such as leaves and roots, metabolic changes during fruit ripening—an essential reproductive phase—are also critical, particularly for nutrient accumulation and defense [26–28]. The genomic features associated with secondary metabolism retained in the *L. caerulea* genome were reflected not only in cold adaptation of the whole plant but also in the regulation of fruit ripening. To investigate this, we measured anthocyanin and iridoid contents at the green maturity and mature stages. Total anthocyanin content increased dramatically from 3.33 mg/100 g at the green maturity stage to 465.84 mg/100 g at full maturity, representing *a* ~140-fold increase, highlighting rapid pigment accumulation. Similarly, iridoid content increased from 12.99 to 33.21 mg/100g, reflecting their co-enrichment during ripening and their known roles in fruit bitterness and medicinal activity (Fig. 5A). Although *L. caerulea* had fewer anthocyanin types compared with kiwifruit, peach, apple, grape, and raspberry, cyanidin-3-O-glucoside consistently dominated its anthocyanin profile (Fig. 5B). This metabolic feature was stable across 14 *L. caerulea* cultivars, highlighting a species-specific metabolic signature likely shaped by genomic background (Fig. 5C).

**Figure 5:**
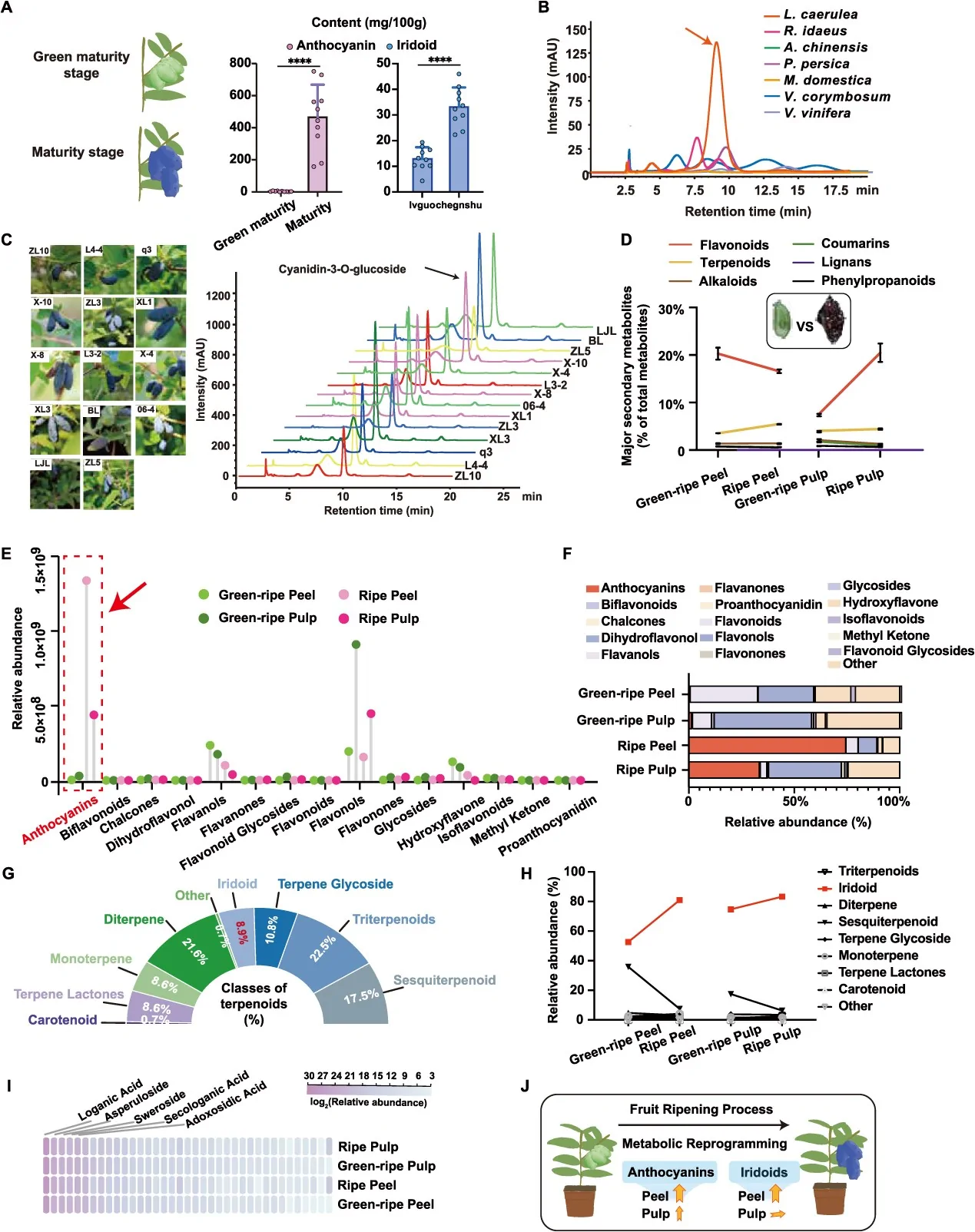
Metabolic dynamics during fruit ripening in *L. caerulea*. (A) Content changes of total anthocyanin and total iridoid in *L. caerulea* fruits at green the maturity stage and the maturity stage. (B) Comparison of anthocyanin profiles between *L. caerulea* and other fruits. (C) Anthocyanin composition analysis in *L. caerulea* cultivars. (D) Major metabolite categories detected in the green unripe peel, mature peel, green unripe flesh, and mature flesh of the fruit. (E) Relative abundance of different flavonoid subclasses in different tissues. (F) Proportional distribution of flavonoid subclasses across peel and pulp at different developmental stages. (G) Classification and proportional distribution of terpenoid metabolites. (H) Relative abundance of different terpenoid subclasses. (I) Heatmap showing the relative abundance of major iridoid metabolites. Data were log_2_-transformed for standardization. The scale bar represents relative metabolite levels. (J) Schematic model illustrating the dynamic changes of anthocyanins and iridoids during the fruit ripening process of *L. caerulea*. Anthocyanins represent the predominant class of metabolites enriched during maturation.

To gain deeper insights, we conducted metabolomic profiling of the peel and pulp at the green maturity and maturity stages. The results showed that flavonoids were the main category, followed by terpenoids. In the peel, the proportions of flavonoid and terpenoid shifted from 20.29%/3.52% to 16.59%/5.42%. In contrast, the pulp showed a sharp rise in flavonoid—from 7.56% to 20.48%—and a moderate increase in terpenoid from 3.88% to 4.41% (Fig. 5D). Among all flavonoid subclasses, anthocyanins had the highest relative content in both tissues (Fig. 5E and F). Terpenoid metabolites were further divided into nine subclasses. Although irodoids represented only 8.9% of the terpenoid type (Fig. 5G), they accounted for the majority of total terpenoids, increasing from 52.60% to 80.98% in the peel and from 74.68% to 83.27% in the pulp (Fig. 5H). The dominant iridoid components included loganic acid, asperuloside, sweroside, secologanic acid, and adoxosidic acid (Fig. 5I). In summary, total anthocyanin levels were significantly enriched in both peel and pulp during ripening, while iridoid accumulations were more pronounced in the peel (Fig. 5J). These metabolic trends are consistent with the genomic features related to secondary metabolism and suggest that *L. caerulea* may enhance both flavor complexity and stress resistance through tightly regulated biosynthetic activity during fruit maturation.

### Roles of *LcF3GT* and *LcSLAS* in regulating anthocyanin and iridoid accumulation during fruit maturation and cold response

Transcriptome analysis revealed that differentially expressed genes (DEGs) during *L. caerulea* fruit ripening were mainly involved in phenylpropanoid biosynthesis, flavonoid biosynthesis, and cutin, suberine and wax biosynthesis pathways. During cold resistance, DEGs were enriched in plant–pathogen interaction, MAPK signaling, and phenylpropanoid biosynthesis pathways (Fig. S5). These transcriptional changes were consistent with the metabolomic results. Based on genomic annotation, we identified key genes involved in anthocyanin biosynthesis: 5 *LcCHS*, 7 *LcCHI*, 1 *LcF3H*, 1 *LcF3′H*, 5 *LcDFR*, 1 *LcANS*, 6 *LcFLS*, and 5 *LcF3GT*. The expression levels of these genes increased substantially during both fruit ripening (1–64-fold) and cold resistance (1–9-fold) (Figs 6A and S6). Correlation analysis showed that their expression positively correlated with anthocyanin compounds such as cyanidin-3-O-arabinoside, cyanidin 3-rutinoside, cyanidin (chloride), cyanidin-3-O-glucoside, and pelargonidin-3,5-O-diglucoside (chloride) (Fig. 6B). Among these, *LcF3GT* stood out as a key enzyme converting unstable anthocyanidins into stable anthocyanins. During ripening, its expression increased ~64-fold in the fruit peel and about 25-fold in the pulp. Under cold stress, its expression was elevated by about 3–6-fold (Fig. 6C). RT-qPCR validated these expression patterns. To verify the role of *LcF3GT* in anthocyanin biosynthesis, we constructed overexpression (OE) and RNA interference (RNAi) vectors and performed transient transformation via *Agrobacterium*-mediated infiltration under both normal temperature (NT) and LT conditions. Under NT, *LcF3GT-*OE significantly boosted both transcript level and anthocyanin content, whereas its RNAi silencing reduced both. Under LT conditions, *LcF3GT*-OE accumulated higher anthocyanins with stronger gene expression, while *LcF3GT*-RNAi showed the opposite trend (Fig. 6D and E). These results confirm LcF3GT as a positive regulator of anthocyanin biosynthesis during both ripening and cold response.

**Figure 6:**
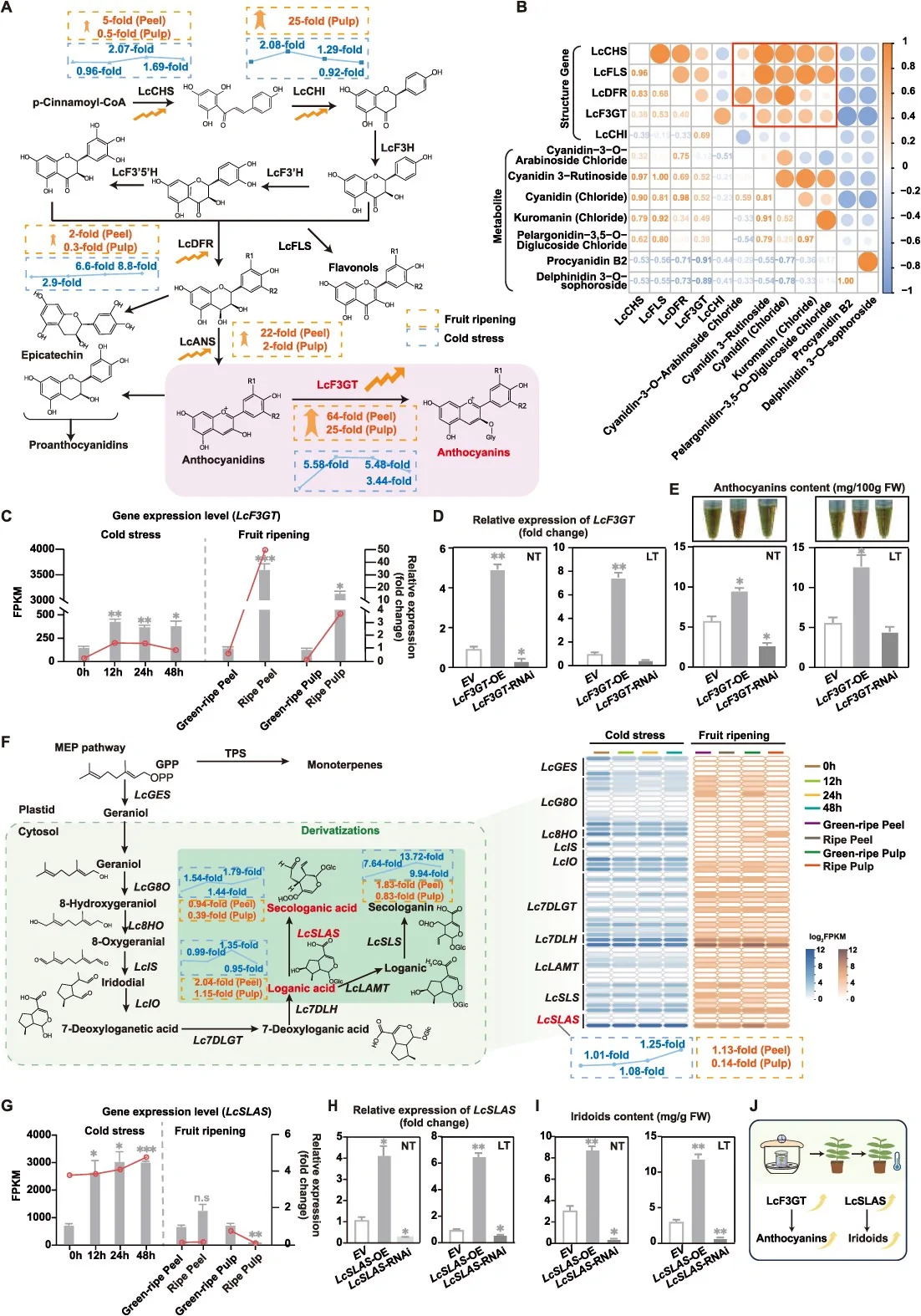
Molecular regulation of anthocyanin and iridoid biosynthesis in *L. caerulea*. (A) The anthocyanin biosynthesis pathway showing fold changes in both metabolites’ and structure enzymes’ gene expression during fruit ripening (fruit ripening process: green-ripe peel, ripe peel, green-ripe pulp, ripe pulp) and cold stress (cold stress process: plants sampled at 0, 12, 24, 48 h). In the pathway panels, LcCHS, LcCHI, LcDFR, LcANS, and LcF3GT refer to gene models evm.model.Chr03.3874, evm.model.Chr07.2164, evm.model.Chr04.2777, evm.model.Chr02.5157, and evm.model.Chr06.3744, respectively. (B) Heatmap of correlation between structural genes involved in anthocyanin biosynthesis and anthocyanin metabolites. (C) Gene expression levels of *LcF3GT* under cold stress and during fruit ripening. (D) Expression levels of LcF3GT in transient overexpression and transient RNAi samples under normal temperature (NT) and low temperature (LT) conditions. (E) Anthocyanin content in *LcF3GT*-OE and *LcF3GT*-RNAi sample under NT and LT conditions. (F) The iridoid biosynthesis pathway showing fold changes of key metabolites and structure enzymes during fruit ripening (fruit ripening process: green-ripe peel, ripe peel, green-ripe pulp, ripe pulp) and cold stress (cold stress process: plants sampled at 0, 12, 24, 48 h). In the pathway panels, LcSLAS refers to gene models evm.model.Chr06.3430. (G) Gene expression levels of *LcSLAS* under cold stress and during fruit ripening. (H) Expression levels of *LcSLAS* in transient overexpression and transient RNAi samples under NT and LT conditions. (I) Iridoid content in LcSLAS transient overexpression and transient RNAi samples under NT and LT conditions. (J) Schematic model illustrating the synergistic regulatory mechanism of the biosynthesis pathways of anthocyanins and iridoid compounds in *L. caerulea*.

Iridoids in *Lonicera* species predominantly exist as secoiridoid glycosides [29]. Based on genomic annotation, we identified core genes involved in iridoid biosynthesis, including 4 *LcGES*, 10 *LcG8O*, 2 *Lc8HO*, 2 *LcIS*, 5 *LcIO*, 12 *Lc7DLGT*, 2 *Lc7DLH*, 8 *LcLAMT*, 6 *LcSLS*, and 3 *LcSLAS*. The biosynthetic pathway begins with GPP, which undergoes dephosphorylation to form linalool, followed by oxidation and cyclization reactions to produce the iridoid skeleton, ultimately leading to the formation of secoiridoid glycosides. As the iridoid biosynthetic pathway in *Lonicera* has not been fully characterized, we integrated known pathways from related species with our metabolomics data. High levels of loganic acid and secologanic acid, alongside low levels of secologanin, allowed us to propose a putative iridoid biosynthetic pathway in *L. caerulea* (Fig. 6F) [30]. We found that loganic acid is a key precursor that is converted by LcSLAS into secologanic acid and by LcSLS into secologanin, both of which are secoiridoid glycosides. During cold stress, the levels of both secologanic acid and secologanin gradually increased. In contrast, during ripening, secologanic acid concentrations in the pulp remained largely unchanged or slightly decreased (~0.94-fold), whereas secologanin levels in the peel increased by ~1.83-fold. Notably, secologanic acid accumulated to much higher levels than secologanin under both ripening and cold stress conditions, underscoring the predominant role of *LcSLAS*. Transcriptomic data showed that *LcSLAS* expression gradually increased during cold tolerance, with a slight increase in the peel (1.13-fold) and a significant decrease in the pulp (0.14-fold). RT-qPCR results were consistent with transcriptomic data. To confirm the function of LcSLAS, we generated *LcSLAS*-OE and *LcSLAS*-RNAi constructs and performed *Agrobacterium*-mediated transient transformation. Under NT conditions, *LcSLAS*-OE promoted iridoid accumulation, while *LcSLAS*-RNAi inhibited it. Under LT conditions, *LcSLAS*-OE further increased iridoid levels, confirming its functional role in enhancing cold resistance via iridoid biosynthesis.

### Molecular regulatory model of *L. caerulea*’s adaptation to cold environments

To explore the regulatory network underlying *L. caerulea*’s environmental adaptation, we constructed a hierarchical model based on correlation and partial correlation analyses [31]. Using gene expression data, we inferred a layered regulatory network to predict both direct and indirect interactions between structural genes and transcription factors (TFs) in secondary metabolism. We first identified the TFs significantly correlated with structural genes during cold treatment and fruit ripening (Fig. 7A and B) and quantified the number of TF families associated with each structural gene (Fig. 7C and D). Under cold conditions, the most prominent regulatory TF families were MYB, ERF, and bHLH. During fruit ripening, the main regulated gene families were MYB, ERF, bHLH, and WRKY. Further analysis revealed that six TFs were predicted to regulate LcF3GT, a key enzyme in anthocyanin biosynthesis, during fruit ripening and cold treatment, while 10 TFs were identified as candidate regulators of LcSLAS, which is central to iridoid biosynthesis (Fig. 7E). Among these, *LcbHLH62* and *LcMYB3R5* showed the strongest positive correlation with *LcF3GT* expression during fruit ripening and were also significantly correlated (*r* = 0.99, *P* < 0.01) in the cold treatment (*r* = 0.8). For LcSLAS, three TFs, including LcMYB15 and LcODO1, were positively correlated (*r* > = 0.8, *P* = 0.01), suggesting their involvement in its regulation (Fig. 7F). To functionally validate these regulatory relationships, we further performed transient overexpression and silencing of these TFs in *L. caerulea* leaves. Compared with the empty vector control, transient overexpression of *LcbHLH62* and *LcMYB3R5* significantly enhanced anthocyanin accumulation, whereas their silencing markedly suppressed anthocyanin biosynthesis (Fig. S7A and B). Similarly, overexpression of *LcMYB15* and *LcODO1* promoted iridoid accumulation, while their silencing suppressed iridoid biosynthesis (Fig. S7C and D). Dual-luciferase reporter assays (Fig. 7G and H) further confirmed that LcbHLH62 directly activated the promoter of *LcF3GT* and that LcMYB15 directly activated the promoter of Lc*SLAS*. These results indicate that LcbHLH62 and LcMYB3R5 act as positive regulators of LcF3GT, driving anthocyanin biosynthesis during both fruit maturation and cold adaptation; LcMYB15 and LcODO1 positively regulate LcSLAS, thereby enhancing iridoid production under similar conditions.

**Figure 7:**
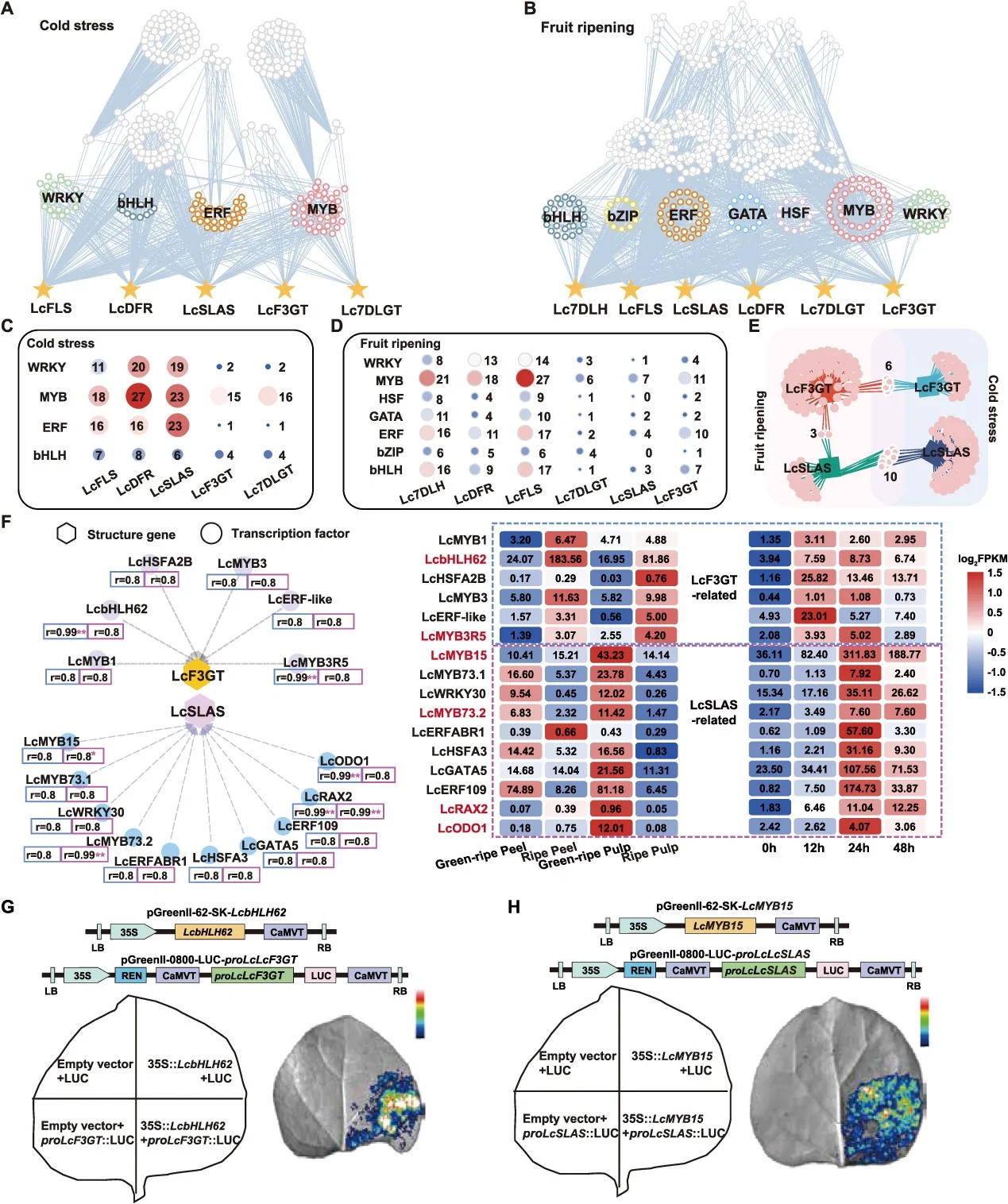
Hierarchical transcriptional regulatory network of anthocyanin and iridoid biosynthesis in *L. caerulea*. (A and B) Predicted hierarchical regulatory networks of key structural enzyme genes involved in anthocyanin and iridoid biosynthesis during (A) cold stress and (B) fruit ripening. (C and D) Distribution of transcription factor (TF) families significantly associated with structural genes under (C) cold stress and (D) fruit ripening. (E) Overlapping TFs regulating anthocyanin and iridoid biosynthesis pathways during fruit ripening and cold stress. (F) The correlation between LcF3GT, LcSLAS, and potential regulatory TFs, as well as the expression levels of these TFs. (G) Activation of LcF3GT promoter by LcbHLH62. (H) Activation of LcSLAS promoter by LcMYB15.

## Discussion

### Metabolic strategy of *L. caerulea* for cold adaptation

*L. caerulea* exhibits exceptional cold tolerance, capable of surviving temperatures as low as −50°C without sustaining damage. It has been commercially cultivated in several cold-prone regions, including the Czech Republic, Russia, Canada, and China. Its fruit is rich in a variety of bioactive compounds with antipyretic, detoxifying, and anti-inflammatory properties and has been used in traditional medicine for thousands of years. Wild populations, particularly those from the Changbai Mountain region—a recognized genetic reservoir in the northern temperate zone—harbor extensive genetic diversity and provide crucial resources for breeding and genetic improvement.

To facilitate deeper molecular investigations of this important species, especially leveraging the rich genetic diversity of its wild populations, we generated a new reference genome for *L. caerulea* from a wild individual collected in the Changbai Mountains. A previous assembly also reported a leaf-derived *L. caerulea* genome [21]. Compared to this prior work, our assembly demonstrates enhanced quality and utility. Our assembly size is 806 Mbp (vs 707.84 Mbp). The estimated heterozygosity in our assembly is 1.26%, compared to 1.63% in their study. Crucially, our BUSCO completeness reaches 99.4%, a substantial improvement over their 92.7%, indicating a more comprehensive gene space. Furthermore, our contig N50 is 69.46 Mbp, showing increased contiguity compared to their 56.96 Mbp; the scaffold N50 is comparable (82.5 vs 83.02 Mbp). These metrics collectively underscore the enhanced quality and utility of our genomic resource, providing a more robust foundation for subsequent functional genomics analyses.

Using this high-quality genome, we integrated transcriptomic and metabolomic analyses to uncover its survival strategy in cold environments (Fig. 8). We identified anthocyanins and iridoids as key metabolites involved in this adaptive response. Under cold stress, two key regulatory modules were activated: the LcODO1/LcMYB15–LcSLAS module promoted the accumulation of iridoids, including secologanic acid, while the LcbHLH62/LcMYB3R5–LcF3GT module regulated anthocyanin biosynthesis. These rapidly inducible pathways likely provide immediate protective functions. Importantly, the capacity to deploy these pathways quickly and robustly in response to temperature drops is itself an adaptive trait. In the wild, *L. caerulea* experiences recurrent cold stress across seasons and years; thus, the repeated activation of these modules during natural temperature fluctuations likely constitutes a core component of the long-term cold adaptation strategy. Interestingly, this metabolic response exhibited clear stage-specific modulation. During autumn and winter, both iridoids and anthocyanins accumulated, with iridoid dominating in quantity—likely reflecting their role in defense. However, during fruit ripening, resource allocation shifts: iridoid content decreased markedly in the pulp, while anthocyanin biosynthesis increased, leading to the characteristic deep blue coloration that not only enhances antioxidant properties but may also promote seed dispersers via animal attraction. In contrast, iridoid levels remained relatively high in the peel, suggesting a continuing role in protective defense [32–34]. Notably, the rich diversity of specific anthocyanin glycosides and iridoid derivatives observed in our metabolomic profiling is closely linked to the expansion of their corresponding structural gene families in the genome. This multicopy retention likely provides the catalytic versatility required for such dynamic metabolic remodeling. Specifically, the predominance of cyanidin-3-O-glucoside in *L. caerulea* fruits suggests a high substrate affinity and specificity of LcF3GT family members toward UDP-glucose and cyanidin aglycones. Although the regulatory functions of key genes such as LcF3GT and LcSLAS were validated via transient transformation and dual-luciferase reporter assays, further direct *in vitro* enzymatic assays are crucial to definitively confirm this proposed substrate specificity and the specific functions of these enzymes within the proposed pathways. Beyond these identified positive regulators, our network analysis has also revealed potential negative regulators. These include TFs resembling *MdbHLH162* in apple [35] and *NtMYB2* in Chinese narcissus [36], which are known negative regulators in other species. Investigating these potential negative regulators will be an important direction for future research, aiming to delineate the complex and finely tuned metabolic regulatory landscape in *L. caerulea*.

**Figure 8:**
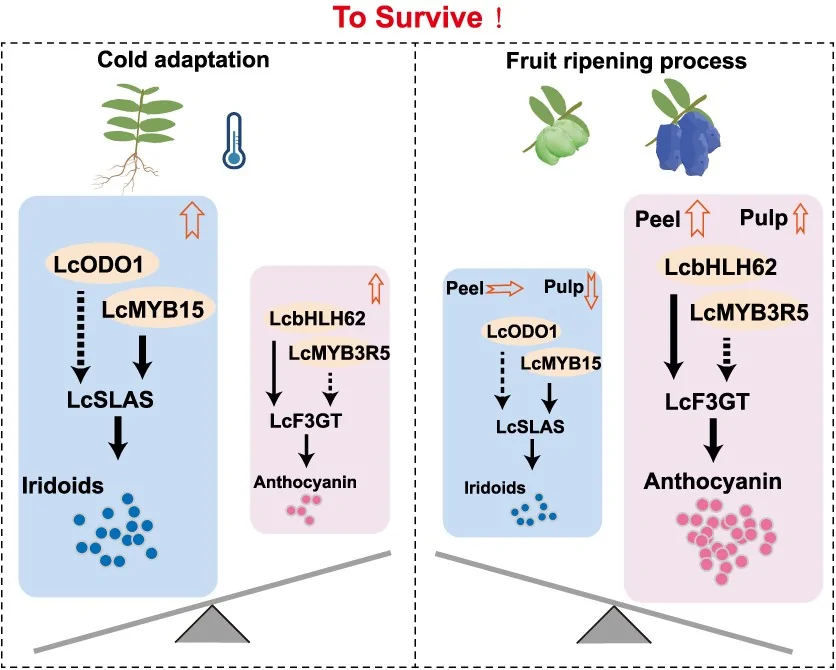
Proposed model of the adaptive survival strategy of *L. caerulea*. This model illustrates the coordinated regulation of key metabolic biosynthesis pathways during its cold adaptation and fruit ripening processes. Cold adaptation module (left): during cold stress, the LcODO1/LcMYB15-LcSLAS pathway drives the robust accumulation of iridoids which serve as the primary chemical defense compounds. Simultaneously, the LcbHLH62/LcMYB3R5-LcF3GT pathway promotes anthocyanin biosynthesis, contributing additional protection through antioxidant activity. Fruit ripening module (right): during ripening, the LcbHLH62/LcMYB3R5-LcF3GT pathway is strongly activated in the pulp to facilitate substantial anthocyanin accumulation, thereby ensuring fruit coloration, nutritional quality, and seed dispersal. In contrast, iridoid content significantly decreases in the pulp while remaining relatively stable in the peel, suggesting a continued defensive role in the outer tissue.

### The diversity of bitter compounds in *L. caerulea* and their potential roles in synergistic defense

The bitterness of plants is widely regarded as a hallmark of chemical defense [37, 38]. Previous studies have identified characteristic bitter compounds in various plant species, such as naringin and neohesperidin in citrus [39], amygdalin in apricot kernels [40], and cucurbitacin in cucumbers [41]. In this study, we systematically explored the bitter metabolite profile of *L. caerulea* by integrating nontargeted metabolomics data with BitterDB database. In addition to iridoids, we identified a range of other bitter compounds, including chlorogenic acid, epicatechin, catechin, isorhamnetin, and other flavonoids, and several amino acids such as L-phenylalanine, L-lysine, and D-phenylalanine. This chemical diversity indicates that the unique bitterness of *L. caerulea* results from a synergistic blend of multiple compound classes rather than the dominance of a single substance.

Bitter metabolites are increasingly recognized as components of plant defense strategies. For example, cucurbitacin C in cucumber can effectively inhibit soil-borne diseases. In *L. caerulea*, we detected a total of 112 bitter metabolites. Among them, multiple bitter metabolites (such as L-Arginine, lonicerin, and genistin) and iridoids accumulated synchronously under cold stress. This coordinated response suggested that these compounds may operate as part of a synergistic chemical defense network against LT conditions. Future research should explore how these bitter components respond to other environmental stresses, whether they interact metabolically, and how their specific tissue-specific accumulation patterns can be used in practical applications. Given the distinct bitterness and unique flavor profile of *L. caerulea*, the present study may offer new insights to guide future breeding efforts aimed at improving fruit flavor. This direction represents a valuable approach for balancing sensory qualities with nutritional and medicinal benefits. A deeper understanding of their ecological and physiological functions will not only clarify the evolutionary basis of bitterness in *L. caerulea* but also support breeding strategies aimed at balancing palatability with resilience. What is particularly noteworthy is that exploiting the distribution of bitter metabolites—for instance, retaining peel-associated bitter components that contribute to stress tolerance while precisely modulating bitterness in the pulp—offers targeted guidance and a theoretical foundation for flavor optimization in L. caerulea fruits.

### Geological influences on the origin and cold adaptation of *L. caerulea*

The geographic distribution and evolutionary origins of plant species are often closely associated with major geological and climatic events. The diversification of the Caprifoliaceae family is temporally aligned with the Cretaceous–Paleogene (K–Pg) mass extinction [42, 43], a period characterized by profound ecological upheaval and subsequent diversification. In this study, divergence time analysis revealed that *L. caerulea* and its close relative *Lonicera* diverged around the Middle Miocene (~17.4 million years ago). This timing coincides with the critical phases of Tibetan Plateau uplift and associated global cooling, events that profoundly reshaped Northern Hemisphere climates and ecosystems [44]. The formation of high-altitude environments likely created geographic and climatic barriers, fostering reproductive isolation and imposing strong selective pressures for cold-resilient traits. We hypothesize that the ancestral *L. caerulea* lineage, subjected to these novel cold environments, underwent adaptive evolution in metabolic pathways, such as those governing anthocyanin and iridoid biosynthesis, that enhanced survival under LT conditions. The gene family expansions and regulatory network modifications we observed in the modern genome may thus reflect historical selection for enhanced cold tolerance. Furthermore, the repeated glacial cycles of the Quaternary period likely created multiple climatic refugia, promoting population differentiation and shaping the genetic architecture of *L. caerulea*. This ‘glacial refugia’ model has also been observed in many other plant species distributed across the Northern Hemisphere [20, 45, 46].

Our study provides fundamental insights into *L. caerulea*’s cold adaptation from a key wild genotype, likely shaped by these geological events. However, to fully test these evolutionary hypotheses and to assess the genetic diversity underlying these adaptive traits across the species’ range and in cultivated varieties, future large-scale population genomic studies, including population resequencing, are crucial. Such research will not only help disentangle the complex interplay between geological events and evolutionary adaptation, deepening our understanding of cold-tolerant plant lineages in northern latitudes, but also bridge the understanding of wild adaptation with practical breeding applications.

## Materials and methods

### Plant materials and sample collection

Wild *L. caerulea* samples were collected from natural populations in the Changbai Mountains, China. For *de novo* genome sequencing and assembly, young leaf tissue was collected from a representative wild individual. To support genome annotation, RNA-seq libraries were generated from multiple tissues, including roots, stems, leaves, and fruits.

Uniformly grown *L. caerulea* seedlings were transferred to growth chambers set at 25, 15, 4, 0, and −4°C, with three biological replicates per treatment. Samples from the 25, 15, 4, and 0°C treatments were used for phenotypic observation. Samples from the 0 and −4°C treatments were used for subsequent transcriptomic and metabolomic analyses. Leaves were sampled from uniformly grown seedlings subjected to the −4°C treatment. Sampling was performed at 0, 12, 24, and 48 h post-treatment. Furthermore, to investigate fruit development, samples of peel and pulp tissues from both the green-ripening and fully ripe stages were collected separately for transcriptomic and metabolomic analyses. All samples were rapidly frozen in liquid nitrogen and stored at −80°C until nucleic acid extraction or metabolite profiling.

### Genome survey and sequencing

A 350-bp short-insert paired-end (PE) library for *L. caerulea* was constructed as follows: genomic DNA was fragmented using the Covaris M220 ultrasonic shearing system. After end repair, A-tailing, adapter ligation, and PCR amplification, sequencing was performed on the Illumina NovaSeq 6000 platform (Illumina, CA, USA) to generate 150-bp PE reads. Raw reads were quality-filtered using custom Perl scripts to remove adapters sequences, reads with >10% unclear bases (Ns), and low-quality reads (>20% of bases with a Phred quality score < 5). Sequencing quality metrics (Q20, Q30, GC content) were analyzed using FastQC v0.11.9. Additionally, 10 000 high-quality reads were randomly selected for BLASTn comparison against the NCBI NT database to assess contamination. k-mer analysis (*k* = 17) was performed using Jellyfish v2.1.3 [47] to estimate genome size, which was calculated by dividing total k-mer count by peak depth with corrections for errors, heterozygosity (based on heterozygous k-mer peak proportion), and repeat content (percentage of k-mers with depth > 1.8 times the main peak).

### *De novo* genome assembly

For *L. caerulea*, a 15–20-kb SMRTbell library was prepared: high-molecular-weight genomic DNA was obtained via G-tube shearing, followed by end repair, adapter ligation, and size selection using the BluePippin system. Sequencing was conducted on the PacBio Sequel II platform (Pacific Biosciences, CA, USA) in the CCS mode. High-quality HiFi reads (length ≥ 500 bp, quality value > 0.99) were generated using CCS v6.3.0. Assembly was performed with Hifiasm v0.15 (parameters: -l 0 to retain haplotype information) [48], and redundant haplotypes were removed. Hi-C library was constructed by fixing tissues and digesting chromatin using MboI, followed by biotin labeling, ligation, and enrichment using streptavidin magnetic beads. Sequencing was performed on the Illumina NovaSeq 6000 platform. Hi-C data were processed with HiCUP v0.7.5 (adapter trimming, BWA-MEM alignment to the contigs, removal of duplicates) [49]. Chromosome-level scaffolding was achieved using ALLHiC v0.9.12 (clustering based on interaction frequency, optimizing order, and orientation via genetic algorithms), followed by manual correction with Juicebox v1.11.08.

### Genome assembly quality assessment

Assembly statistics including total length, contig/scaffold number, maximum length, and N50/N90 values were calculated using QUAST v5.0.2. Completeness was evaluated with BUSCO v5.8.2 [50] and CEGMA v2.5 [51] data. Illumina WGS reads were aligned to the assembled genome using BWA-MEM v0.7.17; mapping ratio, average depth, and coverage were computed with SAMtools v1.15 [52]. SNP detection was performed with BCFtools v1.15 (filters: QUAL < 30, DP < 10, MQ < 40) [52] to assess single-base accuracy. HiC-Pro v3.1.0 [53] and matplotlib v3.5.1 [54] were used to generate Hi-C contact heatmaps (window size: 500 kb) to evaluate chromosomal clustering and intrachromosomal interaction signals.

### Genome annotation

Repeat sequences were annotated through homology-based comparison (RepeatMasker/RepeatProteinMask against RepBase) and *de novo* methods (LTR_FINDER [55], Piler [56], RepeatScout; tandem repeats identified with TRF [57]). Gene structure prediction integrated *de novo*, homology-based, and transcriptome-assisted methods, with results consolidated by EVidenceModeler (EVM) [58]. UTRs and splicing variants were refined using PASA [59]. Functional annotation was performed by aligning predicted genes to NR, Swiss-Prot [60], KEGG, and InterPro databases. Noncoding RNAs were annotated as follows: tRNA genes were identified with tRNAscan-SE [61], rRNA sequences were retrieved by BLAST searches against related species, and miRNA and snRNA genes were predicted using INFERNAL [62] with the Rfam database [63], completing noncoding RNA annotation.

### Comparative genomics analysis

Gene family clustering among blueberry and nine other species was performed using OrthoFinder [64], identifying single-copy, multicopy, and species-specific gene families. Single-copy gene families were aligned with MUSCLE [65] and concatenated into a supermatrix, from which a phylogenetic tree was constructed using RAxML [66] with maximum likelihood. Divergence times were estimated using PAML’s MCMCtree [67]. Gene family expansion and contraction analyses were conducted with CAFÉ [68]. Syntenic blocks were identified with MCScan [69], aligned with MUSCLE [65], and 4DTv values were calculated to infer WGD.

### Physiological measurements

Total anthocyanin content was determined using the pH differential method with KCl-NaAc buffers. Buffer solutions were prepared as follows: 0.025 mol/L KCl (pH = 1) and 0.4 mol/L NaAc (pH = 4.5). Fresh plant tissues (1.0 g) were ground in liquid nitrogen and then extracted in 10 ml of 1% hydrochloric acid in methanol at 4°C in the dark for 24 h. A 1 ml aliquot of the extract was mixed with 4 ml of KCl buffer and 4 ml of NaAc buffer. After thorough mixing, the mixture was kept at 4°C in the dark for 15 min. Absorbance was measured at 510 and 700 nm using a spectrophotometer (UV-2600, Shimadzu).

Dry powder (0.1 g) was extracted in 50 ml of 70% ethanol, which was vortexed to mix uniformly, followed by ultrasonic bath for 45 min. The extracts were then centrifuged at 3500 rpm for 8 min. The supernatant was centrifuged again at 11 300 rpm for 5 min. Then, 1 ml of the supernatant after secondary centrifugation was diluted to an appropriate concentration with 70% ethanol; 1 ml of the diluted sample solution was hydrolyzed with 1 ml of 1 mol/L HCl at 90°C in a water bath for 30 min. After the solutions was cooled to room temperature, 2 ml of 2,4-dinitrophenylhydrazine ethanol was added and reheated to 90°C for 25 min to complete the condensation reaction. After cooling, 1.5 ml of 1 mol/L NaOH in 70% ethanol solution was added, followed by incubation at room temperature for 1 h. Absorbance of the reaction solution was further measured at a wavelength of 462 nm using 70% ethanol as the blank control. Total iridoid content was calculated using the standard curve derived via loganic acid as the standard substance.

Leaf disks (~1 cm^2^) were immersed in 50 ml distilled water at 37°C for 24 h. Electrical conductivity (E1) was measured using a conductivity meter (Leci, DDS-307). The test tube was then heated in boiling water at 100°C for 15 min and cooled to room temperature, and conductivity was measured again (E2). A blank control with distilled water was also tested to measure initial conductivity (E0) and final conductivity after boiling (EL0). Relative electrical conductivity (REC) was calculated as:

### Anthocyanin index measurement

Anthocyanin index (Arildx) was measured using a plant phenotyping imaging system (PlantExplorer PRO). Reflectance at 550 (R550) and 700 nm (R700) was recorded and used to calculate Arildx as follows: Arildx = (1/R550) − (1/R700), where Rλ is reflectance expressed as a fraction (0–1). If the device reported reflectance as a percentage (0–100), it was divided by 100 before calculation. Each sample was measured three times at the same position, and the mean value was used. The instrument was calibrated with the supplied reference tile before measurements. Leaf surfaces were kept dry and consistent orientation was maintained during measurement. Arildx is a relative proxy for anthocyanin content.

### Metabolomic analysis

Metabolomic profiling was performed using ultra-performance liquid chromatography coupled with tandem mass spectrometry (UPLC-MS/MS). Approximately 100 mg of each sample was accurately weighed, flash-frozen in liquid nitrogen, and homogenized into fine powder using a Retsch MM 400 mill at 30 Hz for 1.5 min. The powder was then extracted with 1.2 ml of 70% aqueous methanol. The mixture was vortexed and subjected to an intermittent vortexing procedure (30 s every 30 min, repeated six times) in the dark at room temperature, followed by overnight static extraction. After extraction, the samples were centrifuged at 12 000 rpm for 10 min. The resulting supernatant was collected and filtered through a 0.22-μm microporous membrane, and the filtrate was used for subsequent UPLC-MS/MS analysis. Metabolite identification was achieved by matching the secondary mass spectrometry data against the self-constructed Metware Database of targeted standards, enabling precise annotation based on characteristic fragment ions. For quantification, the multiple reaction monitoring (MRM) mode on a triple quadrupole mass spectrometer was employed. The relative content of each metabolite was calculated using external standard calibration curves, ensuring the reliability and reproducibility of the data.

Fresh fruit samples (1 g fresh weight) were extracted with 4 ml of acidified methanol containing 0.01% HCl. The extraction was performed under dark conditions at 40°C for 60 min using ultrasonic extraction (800 W). The samples were then centrifuged at 14 000 rpm for 10 min. The supernatant was filtered through a 0.22-μm microporous membrane and stored at 4°C until analysis. HPLC analysis was performed using an Agilent 1260 HPLC system (Agilent, San Jose, CA) with a Gemini C18 reverse-phase column (250 × 4.60 mm, 5 μm, Phenomenex, USA). The mobile phase consisted of 0.1% formic acid in water (A) and acetonitrile (B). The gradient elution program was: 0–16 min, 90%–86% A; 16–20 min, 86%–90% A. Flow rate was 1.0 ml/min with an injection volume of 20 μl. Column temperature was maintained at 30°C. Detection was performed at 520 nm. Quantitative analysis was conducted using a standard curve established with the cyanidin-3-O-glucoside standard.

### Transcriptome analysis

Raw RNA-seq reads were processed for quality control using Trimmomatic v0.39 [70]. This step involved the removal of adapter sequences, trimming of low-quality bases (*Q* < 20) from read ends, and discarding of reads with a resulting length below 50 bp, yielding high-quality clean data. The clean reads were then aligned to the *L. caerulea* reference genome using HISAT2 v2.2.1 [71] with default parameters. Gene expression levels were quantified and normalized using the FPKM metric to account for variations in gene length and sequencing depth. Differential expression analysis was performed with the DESeq2 package v1.16.1 [72]. Genes with an adjusted *P*-value (padj) <0.05 and an absolute fold change ≥2 were identified as significantly DEGs. To interpret the biological functions of these DEGs, GO enrichment analysis was conducted using TopGO v2.50.0, and KEGG pathway enrichment analysis was carried out using the clusterProfiler package v4.6.2.

### RNA extraction and RT-qPCR

Total RNA was isolated from samples using the Eastep® Super Plant RNA Kit (Promagna, China). First-strand cDNA was synthesized from the purified RNA using the PrimeScript RT reagent kit with gDNA Eraser (Toyobo, Osaka, Japan) according to the manufacturer’s protocol. The concentration and purity of the synthesized cDNA were assessed using a NanoDrop 8000 spectrophotometer (Thermo Fisher Scientific, Waltham, MA, USA). Quantitative real-time PCR (qRT-PCR) was performed on an Icycler iQ5 system (Bio-Rad, CA, USA) using Fast SYBR Mixture (CWBIO, Beijing, China). Three technical replicates were included for each sample. The β-actin gene was used as an internal reference for data normalization. Primer sequences for qRT-PCR are listed in Table S10. Relative gene expression levels were calculated using the 2^−ΔΔCT^ method.

### Vector construction and genetic transformation

The coding sequence was ligated into the pCAMBIA2300 vector to generate an overexpression construct. For the suppression of gene expression, a specific fragment was cloned in both sense and antisense orientations into the pFGC5941 vector to form an RNAi construct. The resulting plasmids were introduced into *Agrobacterium tumefaciens* strain EHA105 competent cells via electroporation. Positive clones were selected and cultured overnight in LB liquid medium supplemented with 25 mg/L rifampicin and 50 mg/L kanamycin at 28°C with shaking at 180 rpm. Bacterial cells were harvested by centrifugation at 5000 rpm for 10 min, and the pellet was resuspended in MS liquid medium (pH 5.8) to an optical density at 600 nm (OD_600_) of ~0.8. The method was modified based on previous studies [73, 74]. For agroinfiltration, healthy *L. caerulea* seedling leaves were immersed in the bacterial suspension and subjected to a vacuum treatment at −50 kPa for 5 min. After releasing the vacuum, the plants were gently rinsed with distilled water to remove residual bacteria and transferred to sterile potting mix for subsequent analysis. After 48 h of incubation under normal growth conditions, transformed leaf tissues were harvested for metabolite content measurement and gene expression analysis.

### Statistical analysis

All experimental data were statistically analyzed using SPSS (v26, IBM Statistics). Data were presented as ‘mean ± standard deviation (SD)’ based on three biological replicates. Significant differences between two groups were determined by two-tailed unpaired Student’s t-test. A *P*-value <0.05 was considered statistically significant, and a *P*-value <0.01 was considered extremely significant. For multiple comparisons, one-way analysis of variance followed by Tukey’s honest significant difference (HSD) test was performed to assess differences among groups, with adjusted *P*-value < 0.05 set as the threshold for significance.

## Supplementary Material

Web_Material_uhag198

## Data Availability

The genome and transcriptome sequences have been submitted to the National Genomics Data Center (BioProject ID PRJCA053367 and PRJCA042036).
