# Supplementary material for: Adaptive cold-environmental strategies of Lonicera caerulea: synergistic regulation of anthocyanin and iridoid pathways in cold tolerance and fruit ripening
Source: Hortic Res. 2026 May 20;13(10):uhag198. doi: 10.1093/hr/uhag198 (PMC13630454; doi:10.1093/hr/uhag198)
Supplement: Supplementary file 1 [file web_material_uhag198.zip › SupplementFigures.pdf]

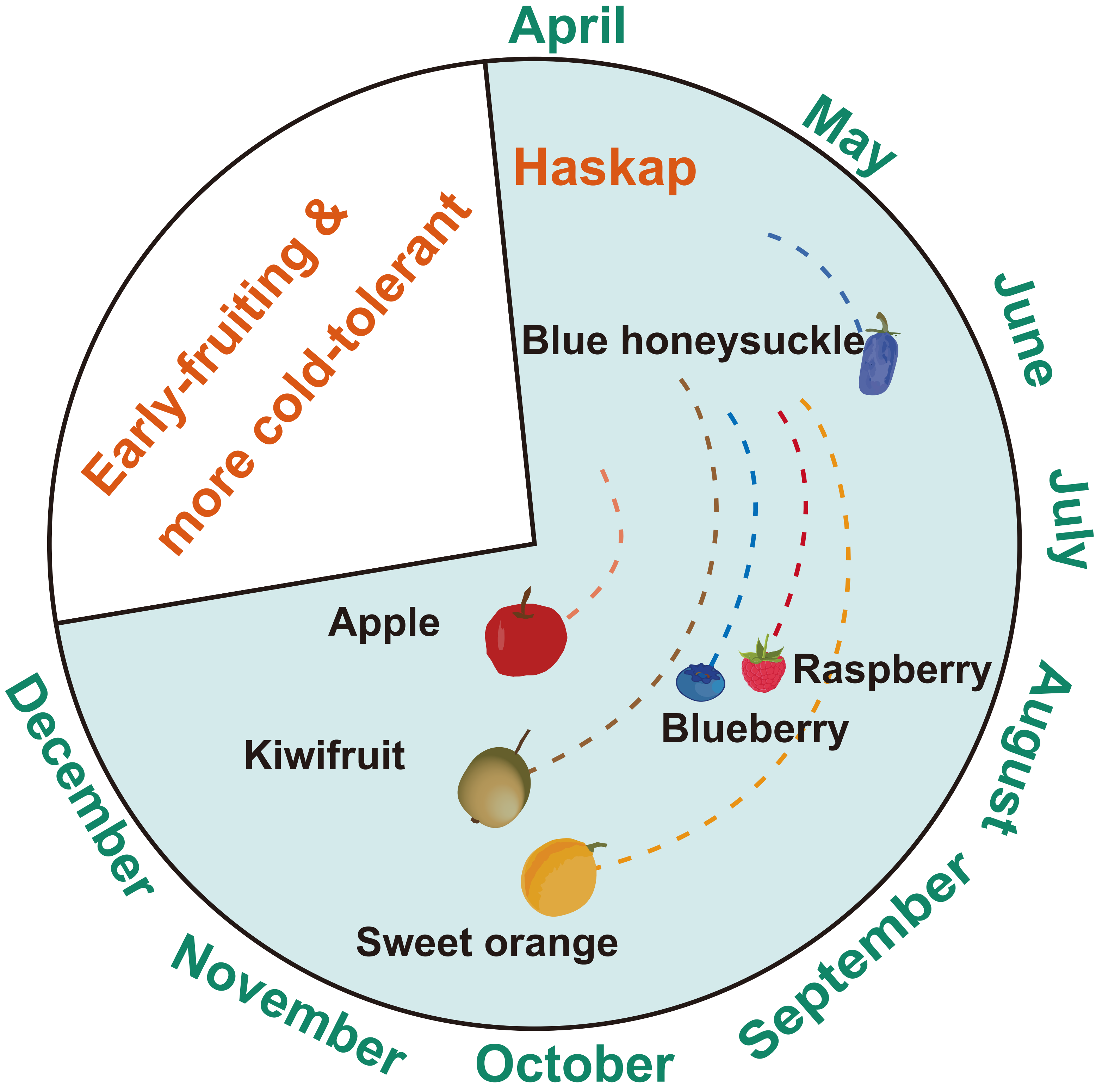
April
May
June
July
August
September
October
November
December
Haskap
Early-fruiting &
more cold-tolerant
Blue honeysuckle
Apple
Raspberry
Blueberry
Kiwifruit
Sweet orange

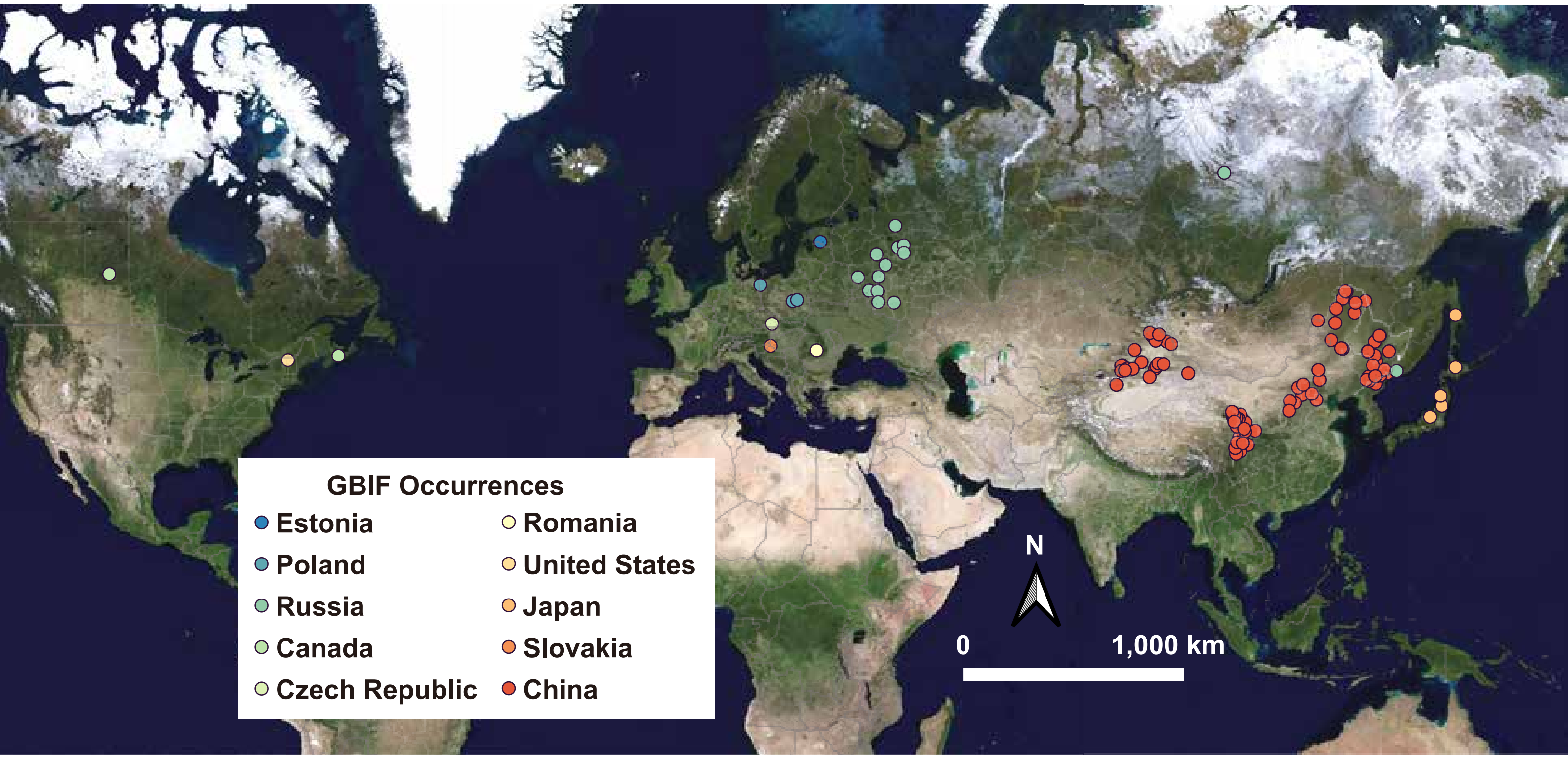
GBIF Occurrences
Estonia
Romania
Poland
United States
Russia
Japan
Canada
Slovakia
Czech Republic
China
N
0
1,000 km

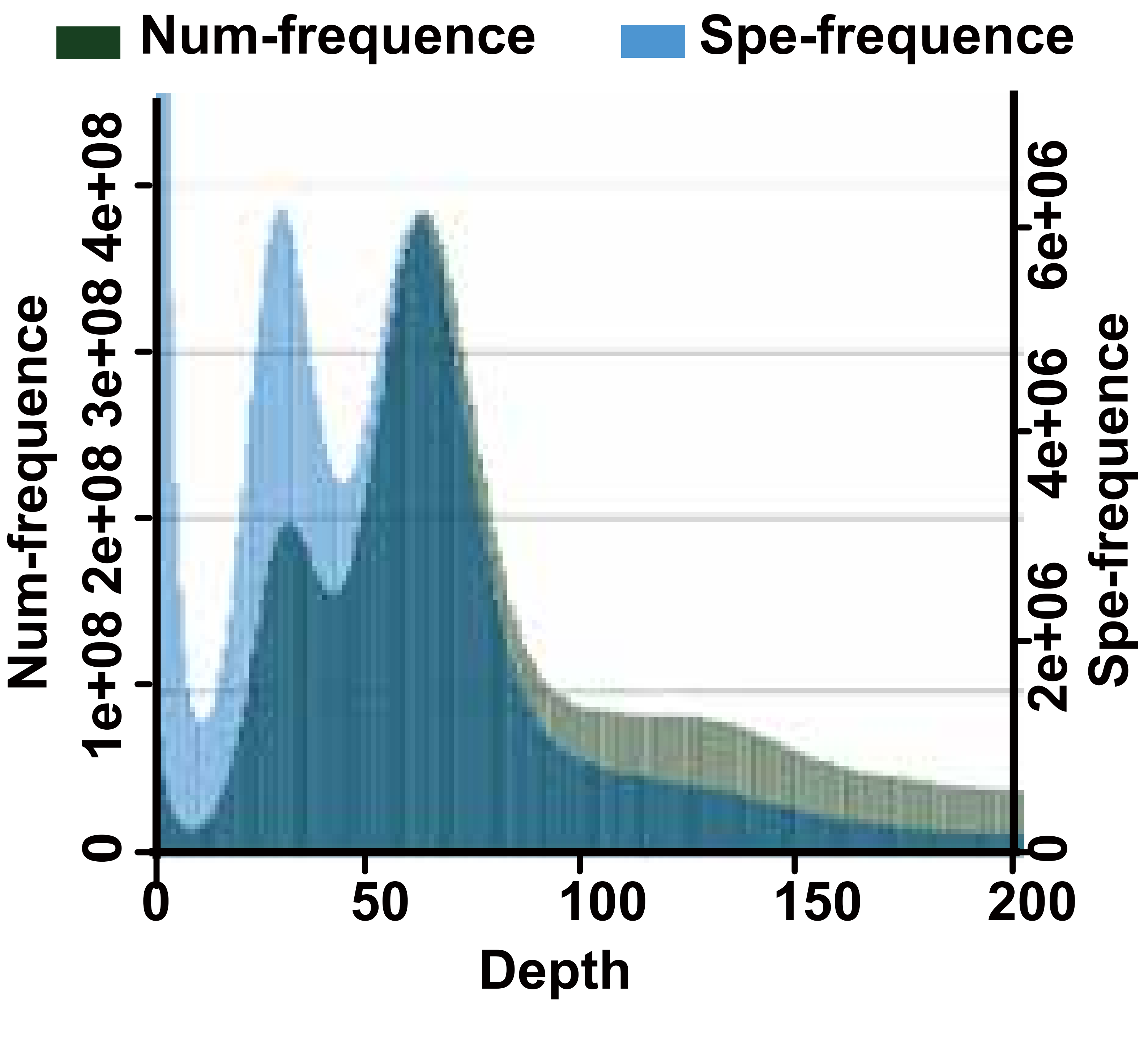

Num-frequence
Spe-frequence
Num-frequence
0
1e+08
2e+08
3e+08
4e+08
Spe-frequence
0
2e+06
4e+06
6e+06
0
50
100
150
200
Depth

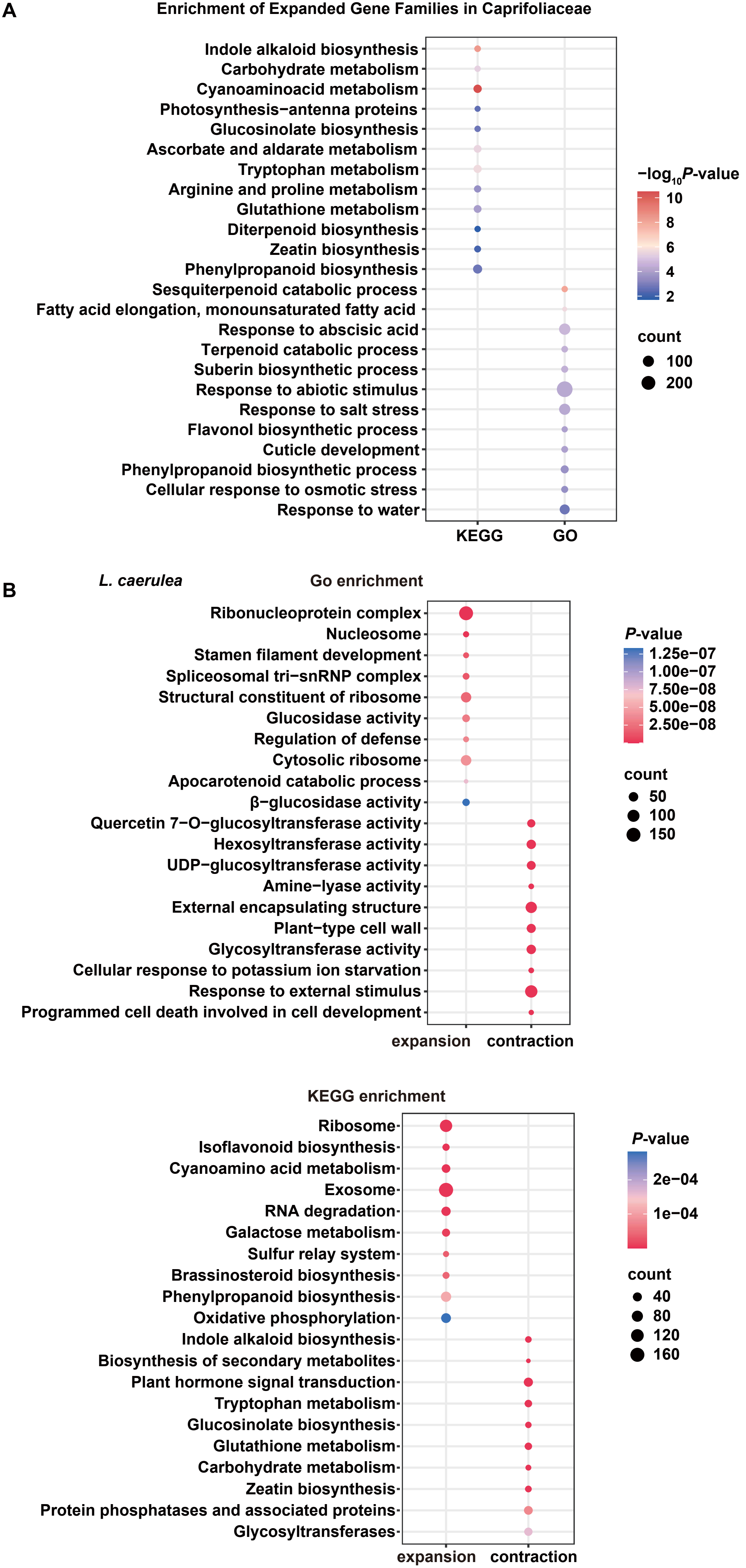
A
Enrichment of Expanded Gene Families in Caprifoliaceae
Indole alkaloid biosynthesis
Carbohydrate metabolism
Cyanoaminoacid metabolism
Photosynthesis−antenna proteins
Glucosinolate biosynthesis
Ascorbate and aldarate metabolism
Tryptophan metabolism
Arginine and proline metabolism
Glutathione metabolism
Diterpenoid biosynthesis
Zeatin biosynthesis
Phenylpropanoid biosynthesis
Sesquiterpenoid catabolic process
Fatty acid elongation, monounsaturated fatty acid
Response to abscisic acid
Terpenoid catabolic process
Suberin biosynthetic process
Response to abiotic stimulus
Response to salt stress
Flavonol biosynthetic process
Cuticle development
Phenylpropanoid biosynthetic process
Cellular response to osmotic stress
Response to water
KEGG
GO
−log10P-value
10
8
6
4
2
count
100
200
B
L. caerulea
Go enrichment
Ribonucleoprotein complex
Nucleosome
Stamen filament development
Spliceosomal tri−snRNP complex
Structural constituent of ribosome
Glucosidase activity
Regulation of defense
Cytosolic ribosome
Apocarotenoid catabolic process
β−glucosidase activity
Quercetin 7−O−glucosyltransferase activity
Hexosyltransferase activity
UDP−glucosyltransferase activity
Amine−lyase activity
External encapsulating structure
Plant−type cell wall
Glycosyltransferase activity
Cellular response to potassium ion starvation
Response to external stimulus
Programmed cell death involved in cell development
expansion
contraction
P-value
1.25e−07
1.00e−07
7.50e−08
5.00e−08
2.50e−08
count
50
100
150
KEGG enrichment
Ribosome
Isoflavonoid biosynthesis
Cyanoamino acid metabolism
Exosome
RNA degradation
Galactose metabolism
Sulfur relay system
Brassinosteroid biosynthesis
Phenylpropanoid biosynthesis
Oxidative phosphorylation
Indole alkaloid biosynthesis
Biosynthesis of secondary metabolites
Plant hormone signal transduction
Tryptophan metabolism
Glucosinolate biosynthesis
Glutathione metabolism
Carbohydrate metabolism
Zeatin biosynthesis
Protein phosphatases and associated proteins
Glycosyltransferases
expansion
contraction
P-value
2e−04
1e−04
count
40
80
120
160

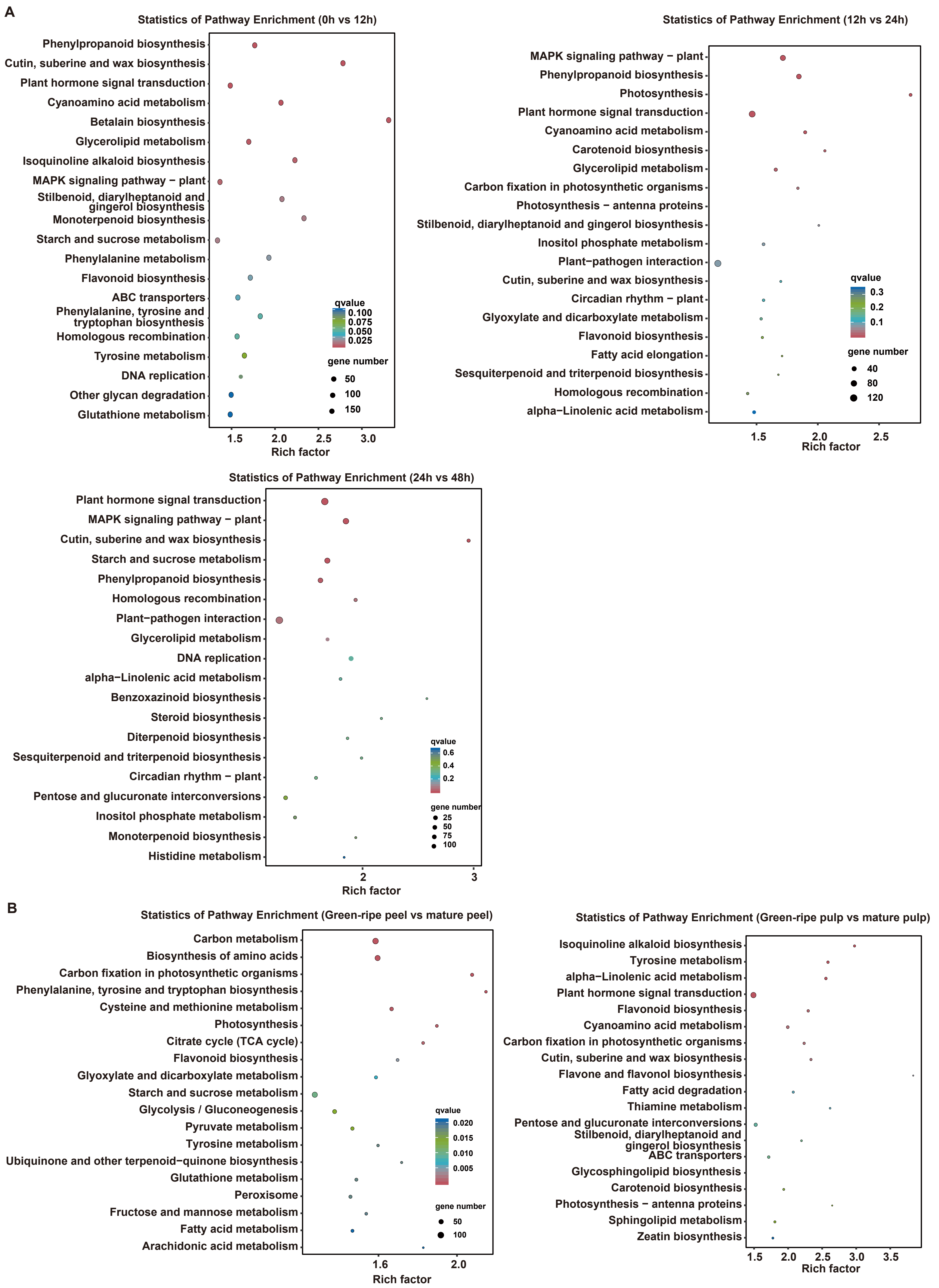
A
Statistics of Pathway Enrichment (0h vs 12h)
Phenylpropanoid biosynthesis
Cutin, suberine and wax biosynthesis
Plant hormone signal transduction
Cyanoamino acid metabolism
Betalain biosynthesis
Glycerolipid metabolism
Isoquinoline alkaloid biosynthesis
MAPK signaling pathway − plant
Stilbenoid, diarylheptanoid and gingerol biosynthesis
Monoterpenoid biosynthesis
Starch and sucrose metabolism
Phenylalanine metabolism
Flavonoid biosynthesis
ABC transporters
Phenylalanine, tyrosine and tryptophan biosynthesis
Homologous recombination
Tyrosine metabolism
DNA replication
Other glycan degradation
Glutathione metabolism
qvalue
0.100
0.075
0.050
0.025
gene number
50
100
150
1.5
2.0
2.5
3.0
Rich factor
Statistics of Pathway Enrichment (12h vs 24h)
MAPK signaling pathway − plant
Phenylpropanoid biosynthesis
Photosynthesis
Plant hormone signal transduction
Cyanoamino acid metabolism
Carotenoid biosynthesis
Glycerolipid metabolism
Carbon fixation in photosynthetic organisms
Photosynthesis − antenna proteins
Stilbenoid, diarylheptanoid and gingerol biosynthesis
Inositol phosphate metabolism
Plant−pathogen interaction
Cutin, suberine and wax biosynthesis
Circadian rhythm − plant
Glyoxylate and dicarboxylate metabolism
Flavonoid biosynthesis
Fatty acid elongation
Sesquiterpenoid and triterpenoid biosynthesis
Homologous recombination
alpha−Linolenic acid metabolism
qvalue
0.3
0.2
0.1
gene number
40
80
120
1.5
2.0
2.5
Rich factor
Statistics of Pathway Enrichment (24h vs 48h)
Plant hormone signal transduction
MAPK signaling pathway − plant
Cutin, suberine and wax biosynthesis
Starch and sucrose metabolism
Phenylpropanoid biosynthesis
Homologous recombination
Plant−pathogen interaction
Glycerolipid metabolism
DNA replication
alpha−Linolenic acid metabolism
Benzoxazinoid biosynthesis
Steroid biosynthesis
Diterpenoid biosynthesis
Sesquiterpenoid and triterpenoid biosynthesis
Circadian rhythm − plant
Pentose and glucuronate interconversions
Inositol phosphate metabolism
Monoterpenoid biosynthesis
Histidine metabolism
qvalue
0.6
0.4
0.2
gene number
25
50
75
100
2
3
Rich factor
B
Statistics of Pathway Enrichment (Green-ripe peel vs mature peel)
Carbon metabolism
Biosynthesis of amino acids
Carbon fixation in photosynthetic organisms
Phenylalanine, tyrosine and tryptophan biosynthesis
Cysteine and methionine metabolism
Photosynthesis
Citrate cycle (TCA cycle)
Flavonoid biosynthesis
Glyoxylate and dicarboxylate metabolism
Starch and sucrose metabolism
Glycolysis / Gluconeogenesis
Pyruvate metabolism
Tyrosine metabolism
Ubiquinone and other terpenoid−quinone biosynthesis
Glutathione metabolism
Peroxisome
Fructose and mannose metabolism
Fatty acid metabolism
Arachidonic acid metabolism
qvalue
0.020
0.015
0.010
0.005
gene number
50
100
1.6
2.0
Rich factor
Statistics of Pathway Enrichment (Green-ripe pulp vs mature pulp)
Isoquinoline alkaloid biosynthesis
Tyrosine metabolism
alpha−Linolenic acid metabolism
Plant hormone signal transduction
Flavonoid biosynthesis
Cyanoamino acid metabolism
Carbon fixation in photosynthetic organisms
Cutin, suberine and wax biosynthesis
Flavone and flavonol biosynthesis
Fatty acid degradation
Thiamine metabolism
Pentose and glucuronate interconversions
Stilbenoid, diarylheptanoid and gingerol biosynthesis
ABC transporters
Glycosphingolipid biosynthesis
Carotenoid biosynthesis
Photosynthesis − antenna proteins
Sphingolipid metabolism
Zeatin biosynthesis
1.5
2.0
2.5
3.0
3.5
Rich factor

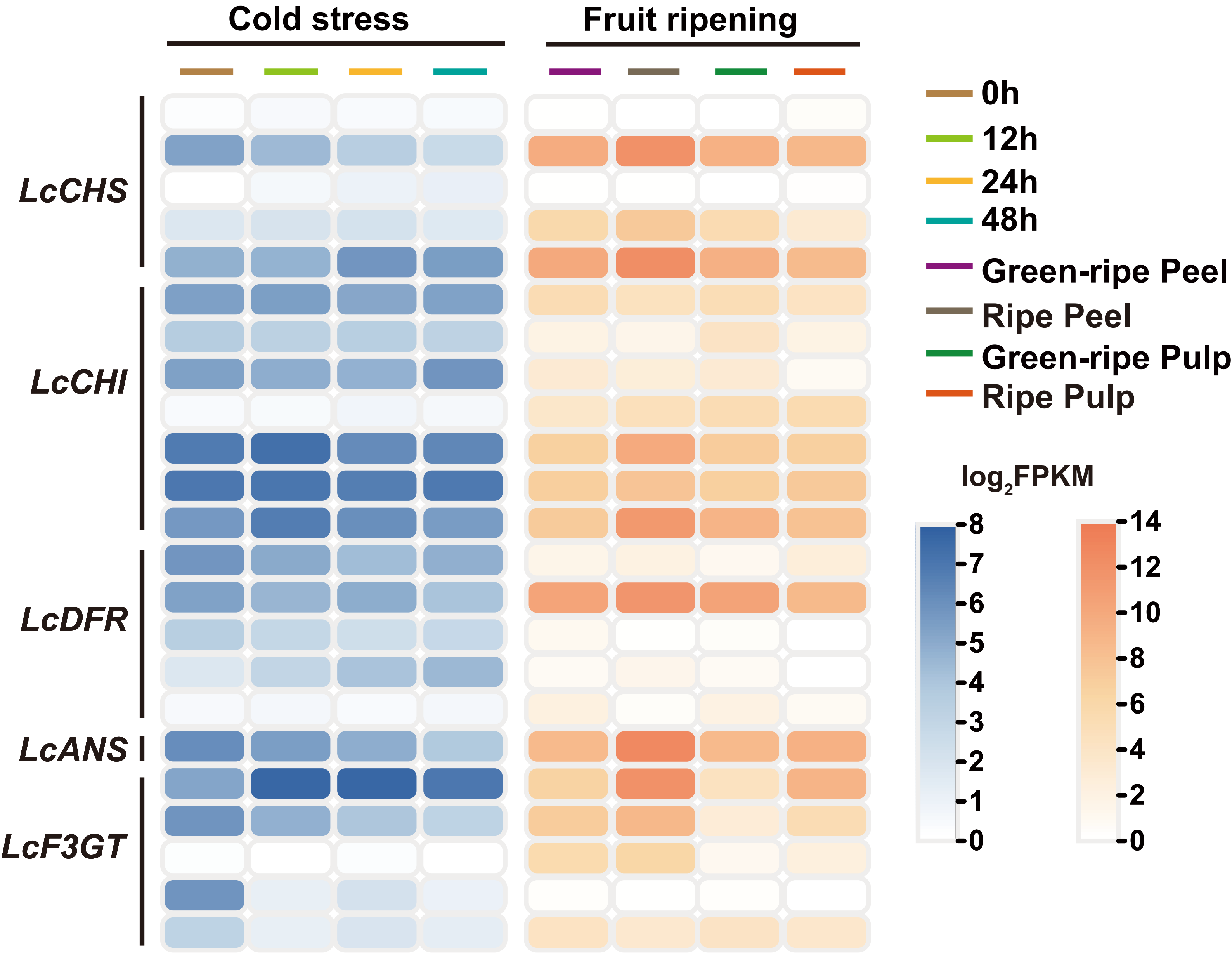

Cold stress
Fruit ripening
LcCHS
LcCHI
LcDFR
LcANS
LcF3GT
0h
12h
24h
48h
Green-ripe Peel
Ripe Peel
Green-ripe Pulp
Ripe Pulp
log2FPKM
8
7
6
5
4
3
2
1
0
14
12
10
8
6
4
2
0

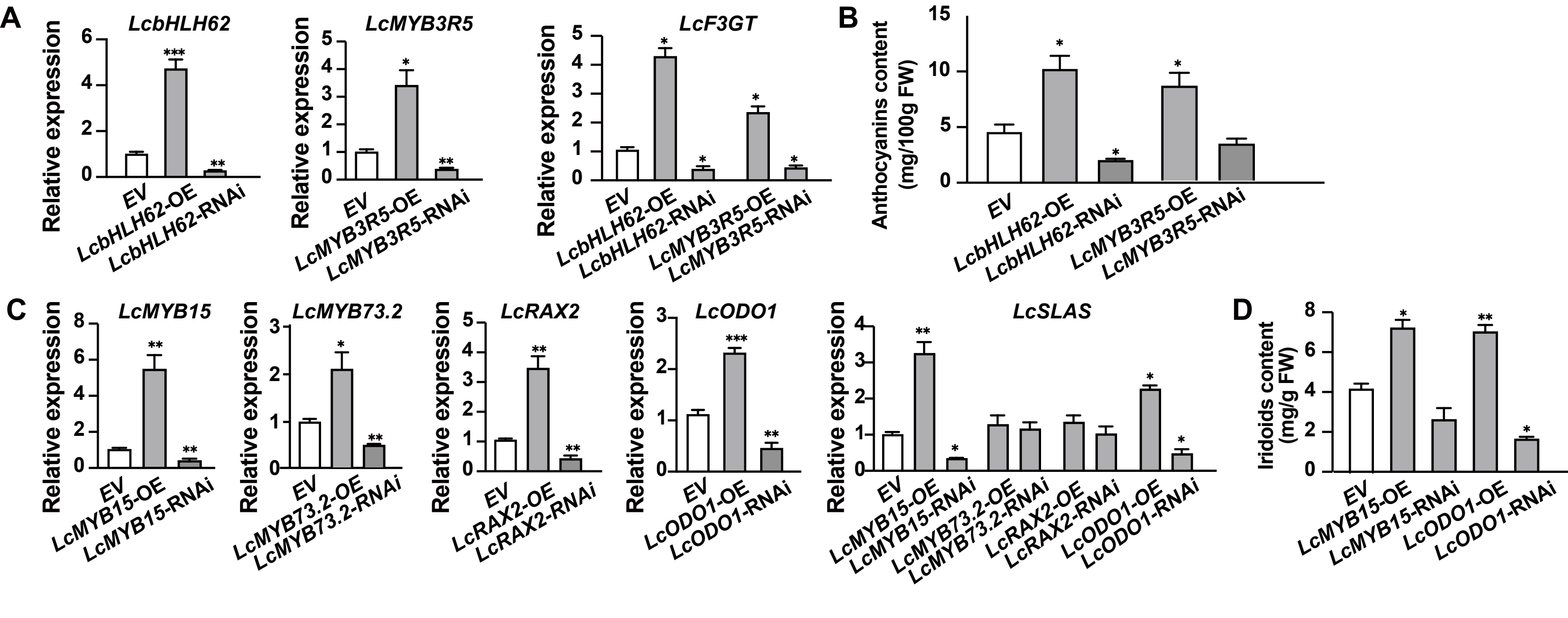

A
LcbHLH62
LcMYB3R5
LcF3GT
Relative expression
EV
LcbHLH62-OE
LcbHLH62-RNAi
LcMYB3R5-OE
LcMYB3R5-RNAi
B
Anthocyanins content (mg/100g FW)
C
LcMYB15
LcMYB73.2
LcRAX2
LcODO1
LcSLAS
LcMYB15-OE
LcMYB15-RNAi
LcMYB73.2-OE
LcMYB73.2-RNAi
LcRAX2-OE
LcRAX2-RNAi
LcODO1-OE
LcODO1-RNAi
D
Iridoids content (mg/g FW)
